# Gut Microbial Metabolite Pravastatin Attenuates Intestinal Ischemia/Reperfusion Injury Through Promoting IL-13 Release From Type II Innate Lymphoid Cells *via* IL−33/ST2 Signaling

**DOI:** 10.3389/fimmu.2021.704836

**Published:** 2021-09-28

**Authors:** Fan Deng, Jing-Juan Hu, Xiao Yang, Qi-Shun Sun, Ze-Bin Lin, Bing-Cheng Zhao, Zhi-Wen Yao, Si-Dan Luo, Ze-Ling Chen, Ying Liu, Zheng-Zheng Yan, Cai Li, Wei-Feng Liu, Ke-Xuan Liu

**Affiliations:** Department of Anesthesiology, Nanfang Hospital, Southern Medical University, Guangzhou, China

**Keywords:** intestinal ischemia/reperfusion injury, metabolites, pravastatin, innate lymphoid cells, intestinal stem cells (ISCs), IL-33 and ST2, IL-13

## Abstract

Intestinal ischemia/reperfusion (I/R) injury is a grave condition with high morbidity and mortality. We previously confirmed that intestinal I/R induces intestinal flora disorders and changes in metabolites, but the role of different metabolites in intestinal I/R injury is currently unclear. Based on targeted metabolic sequencing, pravastatin (PA) was determined to be a metabolite of the gut microbiota. Further, intestinal I/R model mice were established through superior mesenteric artery obstruction. In addition, a co-culture model of small intestinal organoids and type II innate lymphoid cells (ILC2s) was subjected to hypoxia/reoxygenation (H/R) to simulate an intestinal I/R model. Moreover, correlation analysis between the PA level in preoperative feces of patients undergoing cardiopulmonary bypass and the indices of postoperative intestinal I/R injury was carried out. IL-33-deficient mice, ILC2-deleted mice, and anti-IL-13 neutralizing antibodies were also used to explore the potential mechanism through which PA attenuates intestinal I/R injury. We demonstrated that PA levels in the preoperative stool of patients undergoing cardiopulmonary bypass were negatively correlated with the indices of postoperative intestinal I/R injury. Furthermore, PA alleviated intestinal I/R injury and improved the survival of mice. We further showed that PA promotes IL-13 release from ILC2s by activating IL-33/ST2 signaling to attenuate intestinal I/R injury. In addition, IL-13 promoted the self-renewal of intestinal stem cells by activating Notch1 and Wnt signals. Overall, results indicated that the gut microbial metabolite PA can attenuate intestinal I/R injury by promoting the release of IL-13 from ILC2s *via* IL-33/ST2 signaling, revealing a novel mechanism of and therapeutic strategy for intestinal I/R injury.

**Graphical Abstract:**
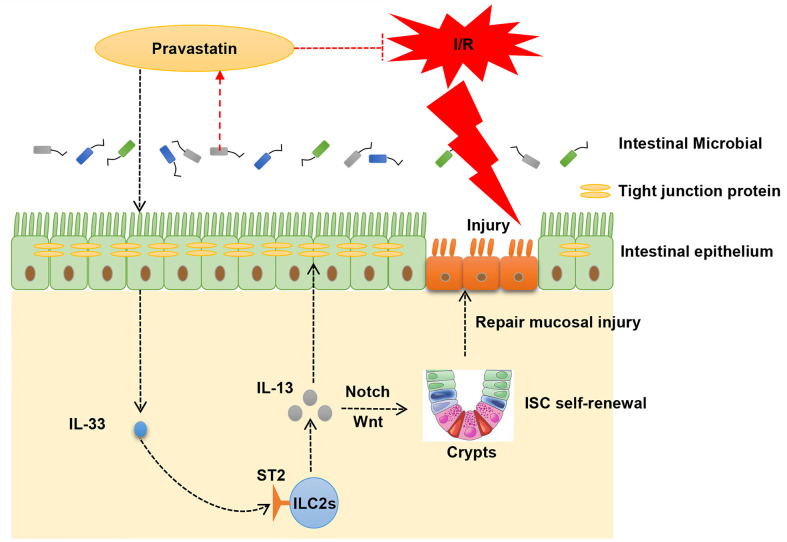


## Introduction

Intestinal ischemia/reperfusion (I/R) injury is a severe condition in some critical clinical settings that is commonly associated with serious infection, shock, and some surgical procedures, including cardiopulmonary bypass (CPB), strangulated ileus, and small bowel transplantation, among others (1). Intestinal I/R not only leads to intestinal injury but also causes damage to multiple extraintestinal organs and even death (2, 3). Currently, the potential mechanisms of intestinal I/R injury have not been fully elucidated, and effective therapeutic strategies need to be further explored.

The human intestinal commensal flora and related metabolites have always been considered factors related to the health of the host (4, 5). We previously confirmed that intestinal I/R induces significant intestinal flora disorders and changes in metabolites and showed that intestinal flora metabolites play an important regulatory role in intestinal I/R injury (6). In this study, we found that pravastatin (PA) is a metabolite of intestinal flora through metabolomic analysis. Statins are mainly derived from secondary metabolites of microbial fermentation and chemical synthesis. One study of various microorganisms revealed some fungi and bacteria that can convert compactin to pravastatin, in addition to strain improvement of *Streptomyces xanthochromogenes* RIA 1098 for enhanced pravastatin production (7). Pravastatin is mainly used to reduce cholesterol levels in patients, and it has been confirmed to promote angiogenesis and resistance to oxidative stress (8, 9), but its role in intestinal I/R injury has not been elucidated.

Interleukin-33 is secreted mainly by non-hematopoietic fibroblast, epithelial, and endothelial cells (10). Interleukin-33 released by epithelial cells activates type II innate lymphoid cells (ILC2s) to mount an immune response through the IL-33 receptor ST2 (11). The widely distributed IL−33 receptor ST2 plays a key role in inflammation and homeostasis by mediating IL-33/ST2 signaling (12). Some statins can promote IL-33 release from myocardial tissue (13). However, whether PA facilitates IL-33 release from intestinal tissue and the role of IL-33/ST2 signaling in intestinal I/R injury remains unclear.

ILC2s are required for the regulation of natural immunity and tissue homeostasis (14). ST2 receptors bind IL-33 on the surface of ILC2s, thereby activating these cells to release IL−13 (15–17). However, the role of ILC2s in intestinal I/R injury is unknown. Interleukin-13 plays an important role in regulating the homeostasis of the intestinal barrier (18). When secreted by ILC2s, IL-13 promotes the self-renewal of intestinal stem cells (ISCs) (19). Intestinal barrier homeostasis and ISC self-renewal are essential for repairing the intestinal mucosa after intestinal I/R damage. However, the role of IL-13 secreted by ILC2s during intestinal I/R injury is unclear.

Based on the aforementioned results, we hypothesized that gut microbial metabolite PA could attenuate intestinal I/R injury by promoting IL-13 release from ILC2s *via* IL−33/ST2 signaling. The present study thus aimed to investigate the effect of PA on intestinal I/R injury and elucidate the mechanism *via* which ILC2s are activated to release IL-13 through IL-33/ST2 signaling and through which IL-13 promotes ISC self-renewal. This study will help to elucidate a novel mechanism of intestinal I/R injury and reveal a new therapeutic strategy for clinical practice.

## Materials and Methods

### Animals

Six-to-eight-week-old specific pathogen-free male C57BL/6 mice were purchased from the animal center of Nanfang Hospital of Southern Medical University (Guangzhou, China). IL-33^−/−^ mice and Rag1^−/−^ mice were purchased from Shanghai Model Organisms Center, Inc, and sterile C57BL/6 mice were purchased from Cyagen Biosciences Company (Suzhou, China). All experimental procedures were carried out in accordance with the National Institutes of Health guidelines and were approved by the local Animal Care and Use Committee of the Nanfang Hospital of Southern Medical University.

### Patient Samples

Patients who need cardiopulmonary bypass (CPB) surgery were used as cases for collecting intestinal I/R samples as previously described (20, 21). The enrollment requirements of all patients were carried out in accordance with the standards we established before (6). The study protocol was approved by the Ethical Committee of Nanfang hospital, Southern Medical University (approval number NFEC-202009-k2-01). All individuals gave informed consent to participate. Finally, a total of 20 patients were enrolled. Blood and fecal samples were collected from the 20 patients before surgery.

Blood samples were collected preoperatively (T0) and at 6 h (T1) and 12 h (T2) after surgery for analyses of intestinal fatty-acid binding protein (IFABP) and citrulline, respectively. The level of IFABP in plasma is a confirmed positive marker (22), while citrulline is a reliable negative biomarker for predicting and diagnosing intestinal I/R injury (23). Fecal samples were collected preoperatively, and the levels of PA were quantified by liquid chromatograph-tandem mass spectrometry (LC-MS/MS). IFABP and citrulline in the plasma samples were measured using a human IFABP ELISA Kit (Bio-Swamp, Wuhan, China) and citrulline ELISA Kit (USCN, Wuhan, China), respectively, at multiple time points (T2-T0) to determine concentration differences. The gastrointestinal complication score of the patient on the second day after surgery was performed according to the acute gastrointestinal injury (AGI) standard described previously (24). The detection of PA, IFABP, citrulline and AGI scores were performed by researchers blinded to the group allocation.

### Intestinal I/R Mouse Model

The mouse model for intestinal I/R injury was established as in our previous study (1, 6). Briefly, the mice were anesthetized with isoflurane. A non-invasive microvascular artery clip was placed on the superior mesenteric artery for 60 min, and the clip was removed for reperfusion for 2 h.

### Extraction and Culture of Organoids and the Establishment of Hypoxia-Reoxygenation (H/R) Models *In Vitro*

The extraction and culture of small intestinal organoids was performed as previously described (6, 25, 26). For the establishment of the organoid H/R model, the organoids were placed in a humid, anaerobic environment at 37°C for 12 h and then placed in an aerobic environment containing 5% CO_2_ in a 37°C incubator for 4 h (6).

### Experimental Design

To explore the protective effect of PA on intestinal I/R injury *in vivo*, the mice were randomly assigned to a sham group that was manipulated in the same manner as the I/R group but without undergoing I/R surgery, an I/R group, or an I/R + PA group that was injected intraperitoneally (i.p.) with 2 mg/kg PA (MedChemExpress) 1 h before inducing intestinal I/R. Organoids were cultured alone or with ILC2s (H/R co-culture model) (**Supplementary Figure S1A**). To explore the protective effect of PA on the H/R injury *in vitro*, the monocultured organoids and the cocultured organoid and ILC2 group were randomly assigned to a normal control (NC that was manipulated in the same manner as the H/R group but without undergoing H/R surgery, an H/R group, and an H/R + PA group in which the organoids were incubated with 10 μmol/L PA 1 h before H/R (**Supplementary Figure S1G**).

To explore the role of IL-33/ST2 signaling in the protective effect of PA against intestinal I/R injury, wild type (WT) mice were randomly divided into the following groups (**Supplementary Figure S2A**): I/R; (2) I/R + PA; (3) I/R + Anti-IL-33, in which WT mice were injected i.p. with 60 μg/kg Anti-IL-33 neutralizing antibody (R&D Systems, Inc., Minneapolis, USA) 2 h before establishing the intestinal I/R model; (4) I/R + PA + Anti-IL-33, in which WT mice were injected i.p. with 60 μg/kg Anti-IL-33 neutralizing antibody 2 h before intestinal I/R and 2 mg/kg PA 1 h before intestinal I/R in mice; (5) I/R + PA + Anti-ST2, in which WT mice were injected i.p. with 2 mg/kg PA and 1.5 mg/kg Anti-ST2 neutralizing antibody (R&D Systems, Inc.) 1 and 2 h, respectively, before establishing the intestinal I/R model in mice. The IL-33^−/−^ mice (IL-33^−/−^) were randomly assigned to I/R and I/R + PA groups.

To explore the role of the IL-33/ST2 axis in the protective effect of PA against H/R injury, the organoids and ILC2s extracted from WT mice were randomly assigned to H/R, H/R + PA, H/R + Anti-IL-33, H/R + PA + Anti-IL-33, and H/R + PA + Anti-ST2 groups. Each co-culture was incubated with 10 μmol/L PA and 10 mg/mL Anti-ST2 neutralizing antibody 1 and 2 h before H/R, respectively. Meanwhile, co-cultured organoids from IL-33^−/−^ mice and ILC2s from WT mice were randomly assigned to H/R and H/R + PA groups (**Supplementary Figure S3A**).

The role that IL-33 plays in the protective effect of ILC2s against intestinal I/R injury was investigated by randomly dividing Rag1^−/−^ mice into an I/R group (Rag1^−/−^ mice were injected i.p. with control rat IgG2b on days −5 and −2 before I/R), an I/R + rmIL-33 group (Rag1^−/−^ mice were injected i.p. with control rat IgG2b on days −5 and −2 before I/R, and 50 μg/kg rmIL-33 (PeproTech, Minneapolis, MN, USA) 2 h before intestinal I/R), an I/R + ILC2^−/−^ group (Rag1^−/−^ mice were injected i.p. with anti-CD90.2 antibody (30-H12; 250 mg per mouse; Biolegend) on days −5 and −2 before I/R surgery), and an I/R + rmIL-33 + ILC2^−/−^ group (Rag1^−/−^ mice were injected i.p. with anti-CD90.2 antibody on days −5 and −2 before I/R surgery and 50 μg/kg rmIL−33, 2 h before I/R; **Supplementary Figure 4A**). To explore the role of IL-33 in the protective effect of ILC2s against organoid H/R injury, organoids extracted from WT mice without ILC2s were randomly assigned to an H/R group and an H/R + rmIL-33 group, in which the organoids were incubated with 10 ng/mL rmIL-33, 2 h before H/R. Meanwhile, co-culture system of organoids and ILC2s extracted from the WT mice were divided into two groups, an H/R or H/R + rmIL-33 group (**Supplementary Figure 5A**). To explore the role of IL-13 in the protective effect of PA or IL-33 against intestinal I/R injury, WT mice were randomly divided into six groups as follows (**Supplementary Figure 6A**): (1) I/R group; (2) I/R + PA group; (3) I/R + rmIL-33 group; (4) I/R + anti-IL-13 group (WT mice were injected i.p. with 400 μg/kg anti-IL-13 neutralizing antibody (R&D Systems, Inc.) 2 h before I/R; (5) I/R + PA + anti-IL-13 group; (6) I/R + rmIL-33 + anti-IL-13 group. To explore the role of IL-13 in the protective effect of PA or IL-33 against organoid H/R injury, the co-cultured organoids and ILC2s extracted from the WT mice were randomly assigned to the following groups: H/R, H/R + PA, H/R + rmIL-33, H/R + anti-IL-13 (co-cultures were incubated with 400 μg/kg anti-IL-13 neutralizing antibody 2 h before H/R), H/R + PA + anti-IL-13, and H/R + rmIL-33 + anti-IL-13 (**Supplementary Figure 7A**).

### Isolation of Cells From Small Intestine Lamina Propria (LP)

Mouse small intestine LP single cells were harvested as described (27). Small intestines of 6–8-week-old male C57BL/6J mice were gently removed from the peritoneal cavity and placed in ice-cold PBS to remove the mesentery, fat, blood vessels, and the Peyer patches. The intestinal lumen was cut along the longitudinal axis and feces and mucus were removed with PBS until the lumen was clear. The intestinal tissues were chopped into 1–2 mm pieces and transferred to cell dissociation medium to remove IEC and intraepithelial lymphocytes. The remaining tissue was transferred to digestion solution and LP single cell suspension was purified with 40% and 80% Percoll solution (Solarbio).

### Flow Cytometric Analysis and Cell Sorting

Mouse small intestine lamina propria (LP) single cells were harvested as described (27). For intracellular cytokine staining, single cell suspensions of LP were stimulated with PMA (25 ng/mL; Sigma-Aldrich, St. Louis, MO, USA) then ionomycin (1 μg/mL; Sigma-Aldrich) for 5 h at 37°C. Brefeldin A (10 μg/mL; Sigma-Aldrich) was added after the first hour of incubation. The cells were sedimented by centrifugation at 600 × g at 4°C, washed with MACS buffer, then nonspecific antibody binding was blocked by incubation with CD16/CD32 antibody (Cat# 14-0161-86, eBioscience, Shanghai, China) for 15 min on ice. The cell surface was stained using APC-Cyanine7-conjugated CD45 Antibody (Cat# A15395, eBioscience), FITC-conjugated mouse Hematopoietic Lineage Antibody Cocktail (Cat# 22-7770-72, eBioscience), and BV421-conjugated Rat Anti-Mouse IL-33R (ST2) antibody (Cat# 566309, BD Biosciences, San Jose, CA, USA). Cells were fixed and permeabilized for intracellular staining using the Foxp3 transcription factor staining buffer set (Cat# 00-5523-00, eBioscience), followed by staining with PE-CF594-conjugated Mouse Anti-GATA3 antibody (Cat# 563510, BD Biosciences), BV786-conjugated Mouse Anti-Mouse RORγt antibody (Cat# 564723, BD Biosciences), and PE-Cyanine7-conjugated IL-13 Monoclonal antibody (Cat# 25-7133-82, eBioscience). Dead cells were excluded by labeling with eFluor 780-conjugated Fixable Viability Dye (Cat# 65-0865-14, eBioscience). Cells were assessed using an LSRFortessa X-20Multidimensional HD Flow Cytometer (BD Biosciences) and the data were analyzed with FlowJo 10 software (BD Biosciences).

We sorted ILC2 in LP single cell suspensions using the MoFlo XDP Ultra-speed flow cell sorting system (Beckman Coulter Inc., Brea, CA, USA). The cell surface was stained using APC-conjugated Rat Anti-Mouse Ly-6A/E (Sca-1; Cat# 17-5981-82, eBioscience), PE-conjugated Anti-Mouse KLRG1 Antibody (Cat# 561621, BD Biosciences), and FITC-conjugated mouse Hematopoietic Lineage Antibody Cocktail (Cat# 22-7770-72, eBioscience). ILC2 were identified as Lin^-^Sca-1^+^KLRG1^+^ (27).

### Culture and Expansion of ILC2, Establishment of Co-Cultured Organoids and ILC2 *In Vitro*

We cultured and expanded ILC2 as described (28). Briefly, inoculate 5,000 cells per well in a round bottom 96-well plate, and add 10% (vol/vol) FBS RPMI complete medium containing 10 ng/ml IL-2 to culture and expand. We use 10 ng/ml IL-2 ^+^ 10 ng/ml IL-25 in 10% (vol/vol) FBS RPMI complete medium to induce ILC2 cell activation and rapid growth. For cell maintenance, we add 10 ng/ml IL-7 to 10% (vol/vol) FBS RPMI complete medium. Change half of the medium every 2 days. The ILC2 and organoids were co-cultured at a ratio of 25:1 in Matrigel.

### Depletion of ILC2s *In Vivo*

For ILC2 depletion, Rag1^−/−^ mice were administered an anti-CD90.2 antibody (30-H12; 250 mg per mouse; Biolegend) or control rat IgG2b on days −5 and −2 before I/R surgery as described (29–32).

### Targeted Metabolomics

Targeted metabolomics (PA measurement) was performed by liquid chromatography-tandem mass spectrometry (LC-MS/MS) as described previously (33). Briefly, cecum samples (100 mg) were dissolved in 900 μL of ice-cold water and extracted *via* sonication in water for 10 min. After ethyl acetate was added and the samples were shaken for 3 min, they were centrifuged at 13000 rpm at 4°C for 10 min. The supernatant was collected and dried with nitrogen and then reconstituted with methanol/ammonium acetate pH4.5 (60:40 v/v) for further computer analysis. The chromatographic separation was performed on the Thermo Scientific Prelude SPLC system, and detection was performed using a Thermo TSQ Vantage triple quadrupole mass spectrometer. Data collection and processing were performed with TraceFinderTM software version 3.3 sp1 (Thermo Fisher Scientific Corp., USA).

### Detection of Organoid Injury by CCK-8 and Lactate Dehydrogenase (LDH) Assays

The levels of organoid injury were assessed with a CCK-8 kit (Dojindo, Shanghai, China) and LDH kit (Nanjing Jiancheng Bioengineering Institute, Nanjing, China). The detection of CCK-8 and LDH was carried out based on the manufacturers’ protocols.

### Hematoxylin-Eosin Staining

Hematoxylin-eosin (HE) was used to evaluate local pathological damage of the small intestine, all procedures were carried out as our previous (6). The pathological scores of intestinal mucosal injury were evaluated by blinded technicians, and were grouped according to the Chiu scoring system (34).

### Immunofluorescence and Immunohistochemistry

Immunofluorescence and immunohistochemistry were performed as previously described (1). Anti-zona occludens 1 (ZO-1) antibody (ab216880, Abcam, Cambridge, MA, USA), anti-Occludin antibody (ab216327, Abcam), anti-IL-33 antibody (ab187060, Abcam), anti-IL-13 antibody (ab106732, Abcam), and anti-Ki67 antibody (ab16667, Abcam) were used to detect protein expression in the intestinal tissue and organoids. Images were captured at 200× with an Olympus immunofluorescence microscope. Quantification of the relative intensity of protein staining was performed by automated image analysis in five randomly chosen 200× fields for each sample.

### RNA Extraction and RT-PCR

RNA was extracted with the TRIzol reagent (Invitrogen, New York, USA). Real-time PCR was performed using the ABI Q5 Real-Time PCR System (Applied Biosystems, Foster City, CA, USA), with the SYBR Green detection protocol (TOYOBO, Tokyo, Japan). The expression of target genes in mice was normalized against that of the housekeeping gene 18S using the 2^-ΔΔCT^ method. The target gene primers are shown in **Supplemental Table 1**.

### Statistical Analysis

Data were analyzed and performed using GraphPad Prism software (version 7.0) by investigators blinded to the group allocation. The results are expressed as the mean ± SEM. Statistical analyses were performed using two-sided Student’s t tests, one-way ANOVA as indicated in the figure legends. *P* values were corrected for Tukey’s test (for comparisons between multiple treatment groups). In addition, the Spearman method was used for correlation statistical analysis. A value of *p* < 0.05 was considered significant.

## Results

### Pravastatin Is a Metabolite of the Intestinal Flora That Is Negatively Correlated With the Degree of Patient Intestinal I/R Injury

**Figure 1:**
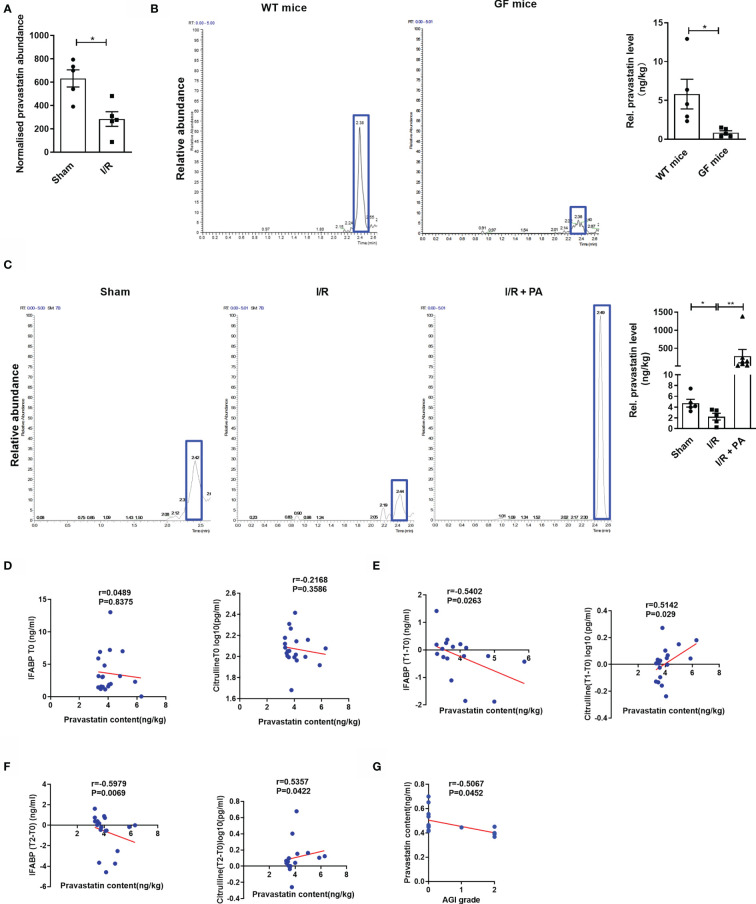
Pravastatin is a metabolite of the intestinal flora and negatively correlated with the degree of patient intestinal I/R injury. **(A)** PA levels in cecum from sham and I/R mice were determined by nontargeted metabolomics (n = 8). **(B)** PA levels in cecum were determined by LC-MS/MS in normal mice and GF mice (n = 6-8). **(C)** PA levels in cecum were determined by LC-MS/MS from sham, I/R and I/R + PA mice (n = 8). **(D–F)** Spearman correlation between the levels of PA in the patients’ preoperative stools and the levels of IFABP and citrulline in the plasma at preoperative (T0), 6 h (T1), or 12 h (T2) after surgery. **(G)** Correlation analysis between the levels of PA in the patients’ preoperative stools and AGI scores of gastrointestinal complications in patients 2 d after surgery.The results are expressed as the mean ± SEM.*p < 0.05, **p < 0.01 by two-tailed Student’s t test **(A–C)**. PA, pravastatin; I/R, ischemia/reperfusion; LC-MS, liquid chromatograph-mass spectrometer; IFABP, intestinal fatty-acid binding protein; AGI, acute gastrointestinal injury.

Differential metabolites represented by PA were screened using t-tests based on a significance level of *p* < 0.05 and a variable importance in projection > 1. Untargeted metabolomic results showed that intestinal I/R caused a significant decrease in PA content in the mouse cecum (**Figure 1A**), and PA had the potential to reduce intestinal I/R damage. Accordingly, whether the gut microbiota contributed to the PA levels was also determined using targeted metabolomic analysis. PA levels in the ceca of normal or germ-free (GF) mice were determined by the external standard method using LC-MS/MS. Levels of PA were significantly decreased in the ceca of GF mice, compared with those in control mice (**Figure 1B**). Levels of PA were significantly reduced in I/R mice, compared with those in sham mice, but PA injected i.p. significantly reversed this trend (**Figure 1C**). These findings showed that the gut microbiota produce PA.

We then collected stool and plasma samples from patients before (T0) CPB surgery and collected plasma samples from patients at 6 (T1) and 12 h (T2) after CPB surgery, determined acute gastrointestinal injury (AGI) scores at 2 d after surgery, and examined the relationships between PA levels and citrulline, intestinal fatty-acid binding protein (IFABP) concentrations, or AGI scores. Results of correlation analysis did not reveal significant relationships between the PA content in stool and plasma levels of citrulline (r = 0.216, *p* = 0.358) or IFABP (r = 0.048, *p* = 0.837) at T0 (**Figure 1D**). In contrast, the PA content in the preoperative stool samples was positively correlated with plasma levels of citrulline, which is a negative marker of intestinal I/R injury, at T1 (r = 0.514, *p* = 0.029) and T2 (r = 0.535, *p* = 0.042) but was negatively correlated with the plasma content of IFABP, a positive marker of intestinal I/R injury, at T1 (r = −0.540, *p* = 0.026) and T2 (r = −0.597, *p* = 0.006), and the AGI score at 2 d (r = −0.506, *p* = 0.045) after surgery (**Figures 1E–G**).

### Pravastatin Reduces Mouse Intestinal I/R Injury and Organoid H/R Injury in Co-Cultured Organoids and ILC2s

**Figure 2:**
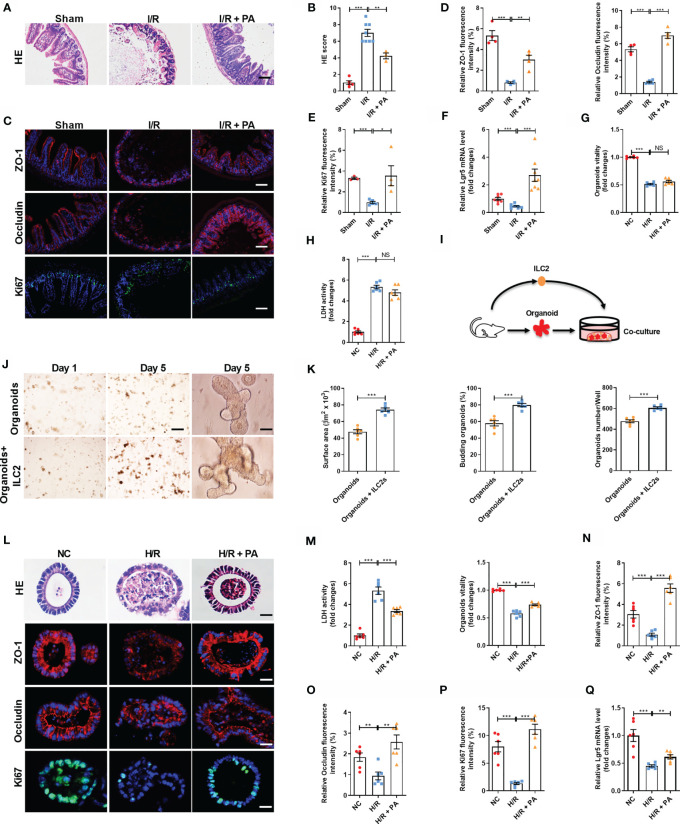
Pravastatin reduced mouse intestinal I/R injury and organoids hypoxia/reoxygenation (H/R) injury in co-cultured organoids and ILC2. **(A, B)** HE staining and pathological damage score of small intestine tissue in sham, I/R and I/R + PA mice, scale bar is 100 μm (n = 8). **(C–E)** The relative protein levels of intestinal tight junction protein ZO-1 and Occludin and cell proliferation marker Ki67 in the ileum were measured by immunofluorescent staining and the relative fluorescence intensity quantification analysis, scale bar is 100 μm (n = 8). **(F)** Relative mRNA level of ISCs self-renewal indicator *Lgr5* was determined by quantitative PCR (n = 8). **(G, H)** Relative organoids viability and LDH levels were detected in organoids cultured alone after H/R (n = 6). **(I)** Schematic representation of co-culture system of organoids and ILC2. **(J)** Brightfield images of organoids without or with group ILC2 at day 1 (scale bar is 400 μm) and day 5 (scale bar is 50 μm), (n = 6). **(K)** Microscopic tracing of organoids to measure surface area, organoids numbers and the percentage of budding organoids at day 5 (n = 6). **(L, M)** HE staining, relative organoids viability and LDH levels were detected in a co-culture system of organoids and ILC2 after H/R, scale bar is 20 μm (n = 6). **(N–P)** The relative fluorescence intensity quantification analysis of ZO-1, Occludin and Ki67 in the organoids, scale bar is 20 μm (n = 6). **(Q)** Relative mRNA level of *Lgr5* in the organoids was measured by quantitative PCR (n = 6). The results are expressed as the mean ± SEM **(B, D, E–H, K, M–Q)**. *p < 0.05, **p < 0.01, ***p < 0.001, NS means No statistically significant difference by one-way ANOVA (Tukey’s test). PA, pravastatin; I/R, ischemia/reperfusion; ISCs, intestinal stem cells; LDH, lactate dehydrogenase; H/R, hypoxia/reoxygenation; ILC2, type II innate lymphoid cells.

We found that PA reduced hematoxylin-eosin staining (HE)-based morphological damage and scores (**Figures 2A, B**). Pravastatin also downregulated the mRNA expression of the inflammatory markers *IL-6* and *IL-1β* (**Supplementary Figures 1B, C**), upregulated mRNA and protein levels of the intestinal mucosal barrier markers *ZO-1* and *Occludin* (**Figures 2C, D** and **Supplementary Figures S1D, E**), and upregulated mRNA and protein levels of proliferation marker *Ki67* (**Figures 2C, E** and **Supplementary Figure S1F**) and the mRNA expression of the ISC marker *Lgr5* (**Figure 2F**). Then, we investigated whether PA exerts protective effects in organoids against H/R injury *in vitro*. Intestinal organoids are 3D systems comprising ISCs, Paneth and enteroendocrine cells, and other intestinal cell types (26). However, unlike the protective effect of PA against intestinal I/R injury *in vivo*, PA did not increase organoid viability and did not reduce LDH release after H/R injury in organoids cultured alone (**Figures 2G, H**). These results implied that the protective effect of PA on intestinal I/R injury is mediated by the interaction between IECs and other immune cells rather than by IECs alone. Considering that type 2 immune cells have an important role in PA effects (35) and that ILC2s with various important functions are abundant in small intestine tissues (27), we investigated whether the protective effect of PA on H/R damage to organoids could be restored in co-cultured ILC2s and organoids (**Figure 2I** and **Supplementary Figure S1G**). The number of co-cultured organoids and the budding ratio were higher and the surface area was larger than those in monocultured organoids (**Figures 2J, K**). Furthermore, PA reduced HE-based morphological damage and levels of LDH released and increased organoid vitality after H/R in the co-culture system (**Figures 2L, M**). Meanwhile, PA upregulated the mRNA and protein levels of *ZO-1* and *Occludin* (**Figures 2L, O** and **Supplementary Figures 1H, I**), the mRNA and protein levels of *Ki67* (**Figures 2L, P** and **Supplementary Figure S1J**), and the mRNA level of *Lgr5* (**Figure 2Q**) in the co-cultures.

### Pravastatin Protects Against Intestinal I/R Injury *via* IL-33/ST2 Signaling

**Figure 3:**
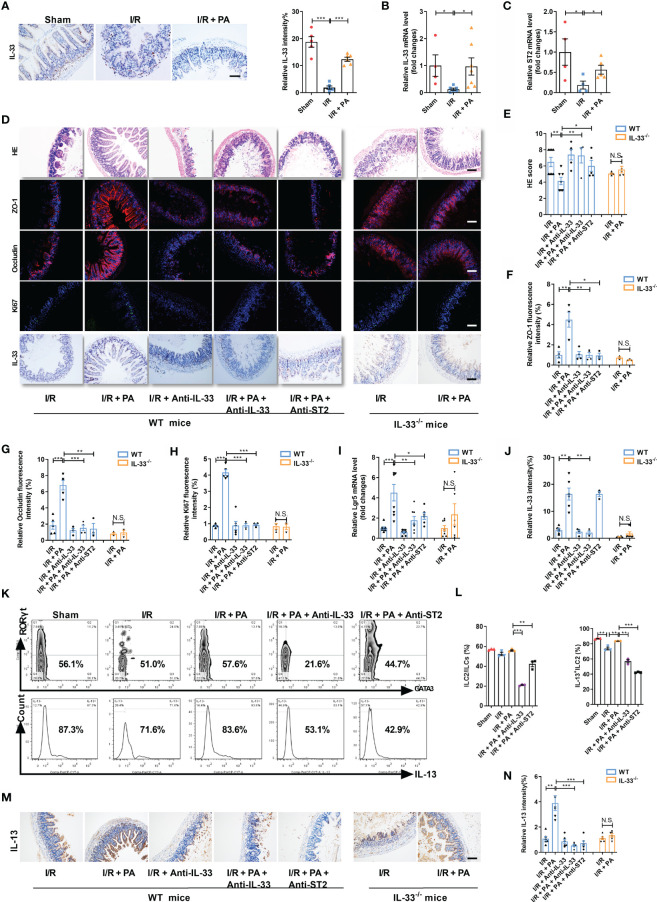
PA protected against intestinal I/R injury *via* an IL-33/ST2 signal. **(A)** IL-33 immunohistochemical staining in the ileum from sham, I/R and I/R + PA mice, scale bar is 100 μm (n = 8). **(B, C)** Relative mRNA levels of IL-33 and IL-33 receptor (ST2) (n = 8). **(D)** HE staining, ZO-1, Occludin and Ki67 immunofluorescent staining and IL-33 immunohistochemical staining in WT mice and IL-33^-/-^ mice, scale bar is 100 μm (n = 8). **(E)** Pathological damage score in the ileum. **(F–H)** The relative fluorescence intensity quantification analysis of ZO-1, Occludin and Ki67 in the ileum. **(I)** The relative mRNA level of *Lgr5* in the ileum was measured by quantitative PCR (n = 8). **(J)** The relative IL-33 intensity quantification analysis in the ileum. **(K, L)** Intestinal lamina propria cells were analyzed by flow cytometry for the ratio of ILC2/ILCs and IL-13^+^ILC2/ILC2 in WT mice (n = 3-4). **(M, N)** IL-13 immunohistochemical staining and intensity quantification analysis in the ileum, scale bar is 100 μm (n = 8). The results are expressed as the mean ± SEM **(B, C, E–J, N)**. *p < 0.05, **p < 0.01, ***p < 0.001, NS means No statistically significant difference by one-way ANOVA (Tukey’s test). PA, pravastatin; I/R, ischemia/reperfusion; ILC2, type II innate lymphoid cells.

Although we confirmed that the protective effect of PA against intestinal I/R injury required the participation of ILC2s, the connection between PA and ILC2s remained unknown. Intestinal I/R damage decreased *IL-33* mRNA and protein levels and *ST2* mRNA levels, effects that were countered by PA (**Figures 3A–C**). These results indicated that IL-33/ST2 signaling might play important role in PA-treated intestinal I/R injury.

Then, neutralizing antibodies against IL-33 (anti-IL-33) or ST2 (anti-ST2) and IL-33^−/−^ mice were used to block IL-33/ST2 signaling (**Supplementary Figure S2A**). The amount of HE-based pathological damage decreased in WT mice administered PA before I/R but not in anti-IL-33- or anti-ST2-treated WT or IL-33^−/−^ mice administered PA (**Figures 3D, E**). The mRNA and protein expression of *ZO-1* and *Occludin* (**Figures 3D, F, G** and **Supplementary Figures S2B, C**), the mRNA and protein expression of *Ki67* (**Figures 3D, H** and **Supplementary Figure S2D**), and the mRNA level of *Lgr5* (**Figure 3I**) were upregulated, whereas the mRNA expression of *IL-6* and *IL-1β* (**Supplementary Figures S2E, F**) was downregulated in PA-treated WT mice but not in anti-IL-33- or anti-ST2-treated WT or IL-33^−/−^ mice administered PA. Meanwhile, the protein expression of IL-33 was upregulated in PA-treated WT mice but not in anti-IL-33-treated WT or IL-33^−/−^ mice administered PA (**Figures 3D, J**). PA also inhibited the reduction in the IL-13^+^ILC2/ILC2 ratio induced by intestinal I/R, but this did not occur in anti-IL-33- or anti-ST2-treated WT mice administered PA (**Figures 3K, L**). Consistent with the results of flow cytometry, the total protein expression of IL-13 in intestinal tissue was upregulated in PA-treated WT mice but not in anti-IL-33- or Anti-ST2-treated WT or IL-33^−/−^ mice administered PA (**Figures 3M, N**).

### Pravastatin Protects Against Organoid H/R Injury *via* IL-33/ST2 Signaling

**Figure 4:**
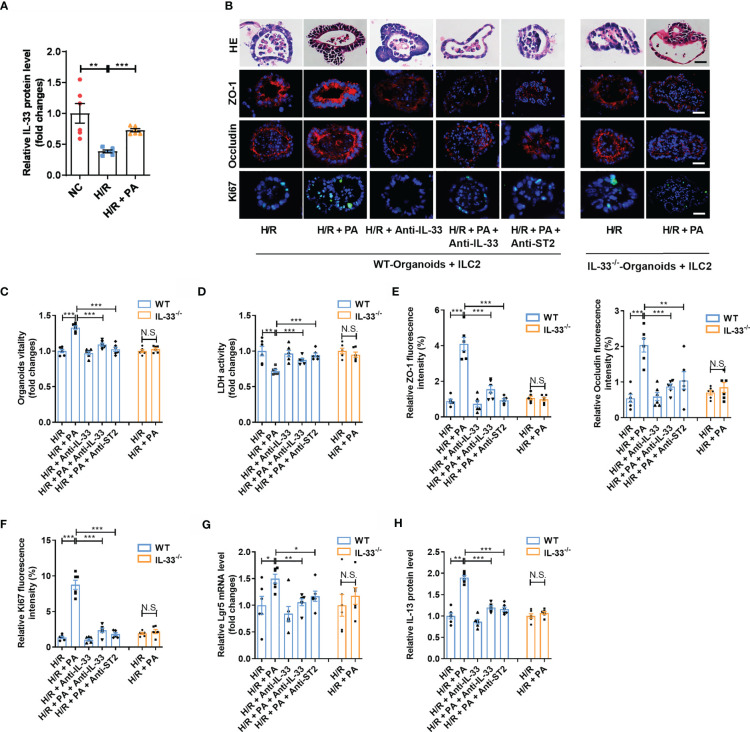
PA protected organoids H/R injury *via* an IL-33/ST2 signal. **(A)** The relative mRNA and protein level of IL-33 in the organoid from WT-Organoids + ILC2. **(B)** HE staining, ZO-1, Occludin and Ki67 immunofluorescent staining in the organoid from WT-Organoids + ILC2, scale bar is 20 μm (n = 6). **(C, D)** Relative organoids viability and LDH levels were detected in a co-culture system of organoids and ILC2 after H/R (n = 6). **(E, F)** The relative fluorescence intensity quantification analysis of ZO-1, Occludin and Ki67 in the organoids (n = 6). **(G)** Relative mRNA level of *Lgr5* in the organoids was measured by quantitative PCR (n = 6). **(H)** The relative protein level of IL-13 in the organoid supernatant determined by ELISA (n = 6). The results are expressed as the mean ± SEM **(A, C–H)**. *p < 0.05, **p < 0.01, ***p < 0.001, NS means No statistically significant difference by one-way ANOVA (Tukey’s test). PA, pravastatin; H/R, hypoxia/reoxygenation; ILC2, type II innate lymphoid cells; LDH, lactate dehydrogenase; WT-Organoids + ILC2, co-cultured WT-organoids and WT-ILC2; IL-33^-/–^Organoids + ILC2, co-cultured IL-33^-/-^ organoids and WT-ILC2.

H/R decreased the expression of IL-33 in the medium in co-cultured organoids and ILC2s, but these changes were counteracted by PA (**Figure 4A**). To further explore the protective mechanism of PA against H/R injury, we added anti-IL-33 or anti-ST2 to the co-cultured ILC2s extracted from WT mice (WT-ILC2s) with organoids from WT mice (WT-organoids) and established co-cultured WT-ILC2 and organoids from IL-33^−/−^ mice (IL-33^−/−^ organoids) to block interactions between IL-33 and ILC2, as well as the subsequent activation of ILC2s (**Supplementary Figure S3A**). Pravastatin inhibited H/R-induced organoid HE-based pathological damage (**Figure 4B**), decreased organoid vitality (**Figure 4C**), and increased LDH release (**Figure 4D**) in co-cultured WT-organoids and WT-ILC2s, but these effects were eliminated by anti-IL-33 or anti-ST2, and no effects were found in co-cultured IL-33^−/−^ organoids and WT-ILC2s. Pravastatin also suppressed the decrease in the mRNA and protein levels of *ZO-1* and *Occludin* (**Figure 4B, E** and **Supplementary Figures S3B, C**), the mRNA and protein levels of *Ki67* (**Figures 4B, F** and **Supplementary Figure S3D**), and the mRNA level of *Lgr5* (**Figure 4G**) caused by H/R in co-cultured WT-organoids and WT-ILC2s, but these effects were eliminated by anti-IL-33 or anti-ST2, and no effects were found in co-cultured IL-33^−/−^ organoids and WT-ILC2s. In addition, PA suppressed the decrease in the protein levels of IL-13 caused by H/R in co-cultured WT-organoids and WT-ILC2s, but these effects were eliminated by anti-IL-33 or anti-ST2, and no effects were found in co-cultured IL-33^−/−^ organoids and WT-ILC2s (**Figure 4H**).

### Depletion of ILC2s Abolishes the Protective Effect of IL-33 on Intestinal I/R Injury and Organoid H/R Injury

**Figure 5:**
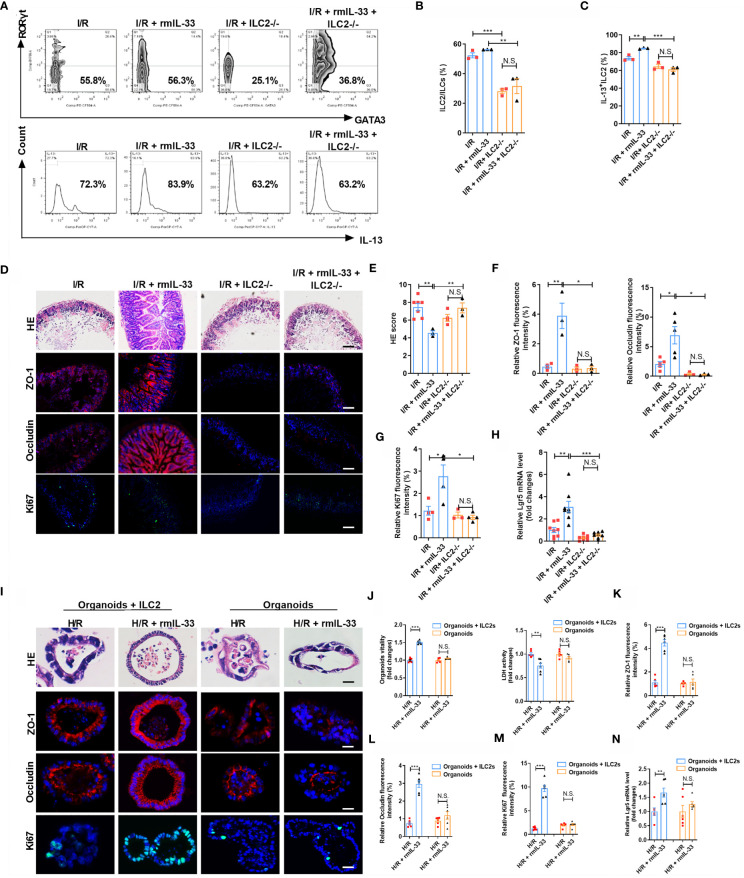
Depletion of ILC2 abolished the protective effect of IL-33 on intestinal I/R injury and organoids H/R injury. **(A–C)** Intestinal lamina propria cells were analyzed by flow cytometry for the ratio of ILC2/ILCs and IL-13^+^ILC2/ILC2 in Rag1^-/-^ mice (n = 3-4). **(D)** HE staining, ZO-1, Occludin and Ki67 immunofluorescent staining in Rag1^-/-^ mice, scale bar is 100 μm (n = 8). **(E–G)** Pathological damage score and the relative fluorescence intensity quantification analysis of ZO-1, Occludin and Ki67 in the ileum (n = 8). **(H)** The relative mRNA level of *Lgr5* in the ileum was measured by quantitative PCR (n = 8). **(I)** HE staining, ZO-1, Occludin and Ki67 immunofluorescent staining in WT-organoid cultured with and without ILC2, scale bar is 20 μm (n = 6). **(J)** Relative organoids viability and LDH levels were detected in WT-organoid cultured with and without ILC2 after H/R (n = 6). **(K–M)** The relative fluorescence intensity quantification analysis of ZO-1, Occludin and Ki67 in the organoids (n = 6). **(N)** Relative mRNA level of *Lgr5* in the organoids was measured by quantitative PCR (n = 6). The results are expressed as the mean ± SEM **(E–H, J–N)**. *p < 0.05, **p < 0.01, ***p < 0.001 by one-way ANOVA (Tukey’s test) **(B–H)**. *p < 0.05, **p < 0.01, ***p < 0.001, NS means No statistically significant difference by two-tailed Student’s t test **(J–N)**. PA, Pravastatin; I/R, ischemia/reperfusion; H/R, hypoxia/reoxygenation; ILC2, type II innate lymphoid cells; LDH, lactate dehydrogenase.

Interleukin-33 is an important ILC2 activator, but the modulation of ILC2s under intestinal I/R injury and the role of ILC2s in the protective effect of IL-33 on intestinal I/R injury remain unclear. We depleted ILC2s using a previously described antibody depletion method to confirm the role of ILC2s in IL-33-mediated protection against intestinal I/R injury (**Supplementary Figure S4A**) (29–32). Recombinant murine IL-33 (rmIL-33) increased the ratio (%) of ILC2/ILCs and IL-13^+^ILC2/ILC2 but not in ILC2-deleted mice (**Figures 5A–C**). Compared with that in the I/R group, rmIL-33 decreased the HE-based morphological damage but not in ILC2-deleted mice (**Figures 5D, E**). Furthermore, the mRNA and protein expression of *ZO-1* and *Occludin* was upregulated (**Figures 5D, F** and **Supplementary Figures S4B, C**), the mRNA and protein levels of *Ki67* (**Figures 5D, G** and **Supplementary Figure S4D**) and the mRNA level of *Lgr5* were elevated (**Figure 5H**), and mRNA levels of *IL-6* and *IL-1β* (**Supplementary Figures S4E, F**) were downregulated in mice administered rmIL-33, but the deletion of ILC2s abolished all of these changes.

To explore the role of ILC2 in protection against H/R-induced organoid injury conferred by IL-33, we added rmIL-33 to organoids cultured with and without ILC2s (**Supplementary Figure S5A**). Recombinant murine IL-33 did not affect HE-based pathological damage (**Figure 5I**), organoid vitality (**Figure 5J**), or LDH release (**Figure 5J**) in WT-organoids cultured without ILC2s but increased organoid vitality and decreased HE-based pathological damage and LDH release in WT-organoids co-cultured with ILC2s (**Figures 5I, J**). Recombinant murine IL-33 upregulated the mRNA and protein expression of *ZO-1* and *Occludin* (**Figures 5I, K, L** and **Supplementary Figures S5B, C**), the mRNA and protein expression of *Ki67* (**Figures 5I, M** and **Supplementary Figure S5D**), and the mRNA level of *Lgr5* (**Figure 5N**) in co-cultured WT-organoids and ILC2 systems but not in WT-organoids cultured without ILC2s. These results indicate that the protection of organoids by IL-33 during H/R injury requires ILC2 participation.

### The Protective Effect of PA/IL-33 to Alleviate Intestinal I/R Injury Is Mediated by IL-13 Released From ILC2s

**Figure 6:**
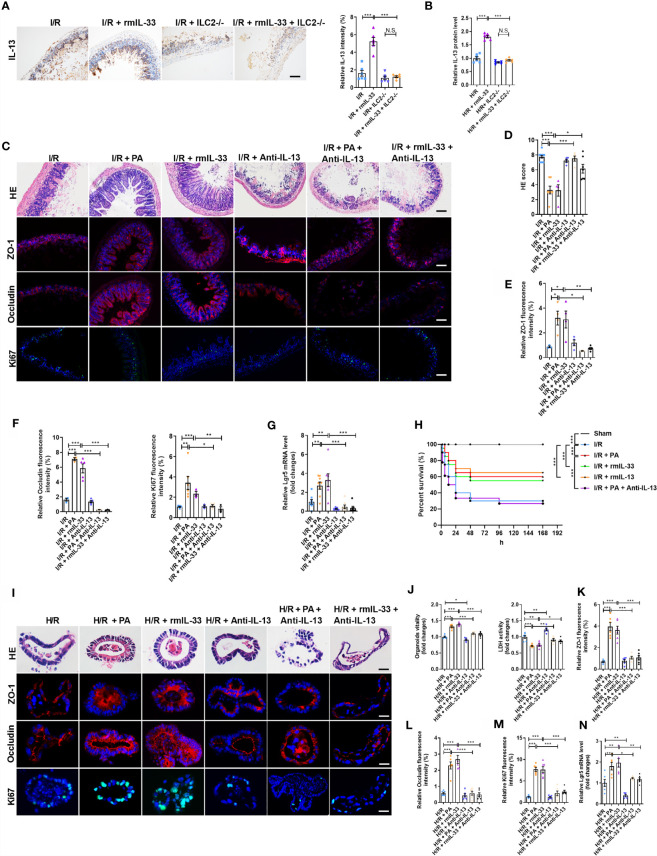
The protection of PA/IL-33 to alleviate intestinal I/R injury was mediated by IL-13 released from ILC2. **(A)** IL-13 immunohistochemical staining and intensity quantification analysis in the ileum, scale bar is 100 μm (n = 8). **(B)** The relative protein level of IL-13 in the organoid supernatant determined by ELISA in WT-organoid cultured with and without ILC2 (n = 6). **(C)** HE staining, ZO-1, Occludin and Ki67 immunofluorescent staining of ileum in WT mice, scale bar is 100 μm (n = 8). **(D–F)** Pathological damage score and the relative fluorescence intensity quantification analysis of ZO-1, Occludin and Ki67 in the ileum (n = 8). **(G)** The relative mRNA level of *Lgr5* in the ileum was measured by quantitative PCR (n = 8). **(H)** Changes in survival rate have been shown. Mice were submitted to ischemia of SMA for 60 min, and survival was monitored on day 7 after reperfusion (n = 20). **(I)** HE staining, ZO-1, Occludin and Ki67 immunofluorescent staining in a co-culture system of organoids and ILC2 after H/R, scale bar is 20 μm (n = 6). **(J)** Relative organoids viability and LDH levels were detected in a co-culture system of organoids and ILC2 after H/R (n = 6). **(K–M)** The relative fluorescence intensity quantification analysis of ZO-1, Occludin and Ki67 in the organoids (n = 6). **(N)** Relative mRNA level of *Lgr5* in the organoids was measured by quantitative PCR (n = 6). The results are expressed as the mean ± SEM **(B, D–G, J–N)**. *p < 0.05, **p < 0.01, ***p < 0.001, NS means No statistically significant difference by one-way ANOVA (Tukey’s test) and Log-Rank test **(H)**. PA, pravastatin; I/R, ischemia/reperfusion; H/R, hypoxia/reoxygenation; ILC2, type II innate lymphoid cells; SMA, superior mesenteric artery; LDH, lactate dehydrogenase.

Studies have shown that activated ILC2s release large amounts of IL-13 (29, 36). Recombinant murine IL-33 increased the protein levels of IL-13 after I/R but not in ILC2-depleted mice (**Figure 6A**). Recombinant mIL-33 increased the protein levels of IL-13 in the WT-organoids co-cultured with ILC2s after H/R but not in WT-organoids cultured without ILC2s (**Figure 6B**). These results indicated that IL-33-activated ILC2s are an important source of IL-13 in the intestine during I/R.

We next investigated the role of IL-13 in the protective effect of PA or IL-33 on intestinal I/R injury using an anti-IL-13 neutralizing antibody (anti-IL-13; **Supplementary Figure S6A**). Pravastatin or rmIL-33 decreased the HE-based pathological damage after I/R, and these effects were abolished by anti-IL-13 (**Figures 6C, D**). The mRNA and protein levels of *ZO-1* and *Occludin* were upregulated (**Figures 6C, E, F** and **Supplementary Figures S6B, C**), the mRNA and protein levels of *Ki67* (**Figure 6C, F** and **Supplementary Figure S6D**) and the mRNA level of *Lgr5* (**Figure 6G**) were elevated, and the mRNA expression of *IL-6* and *IL-1β* was downregulated (**Supplementary Figures S6E, F**) in PA- or rmIL-33-treated mice, but anti-IL-13 abrogated all of these changes induced by PA or rmIL-33. Pravastatin, rmIL-33, and rmIL-13 increased the survival rate of mice after I/R, but the effect of PA was abolished by anti-IL-13 (**Figure 6H**).

To further determine the role of IL-13 released by ILC2 in the protective effects of PA/IL-33 against H/R-induced organoid injury, we added the anti-IL-13 antibody to the co-cultured WT-organoids and WT-ILC2s in the presence of PA or rmIL-33 (**Supplementary Figure S7A**). The organoid vitality was higher and the amount of HE-based pathological damage and levels of released LDH were lower in the PA- or rmIL-33-treated cultures than in the untreated co-cultures during H/R (**Figures 6I, J**). Meanwhile, PA or rmIL-33 upregulated the mRNA and protein levels of *ZO-1* and *Occludin* (**Figures 6I, K, L** and **Supplementary Figures S7B, C**), the mRNA and protein levels of *Ki67* (**Figures 6I, M** and **Supplementary Figure S7D**), and the mRNA level of *Lgr5* in the co-cultures (**Figure 6N**). However, the protective effects of PA or IL-33 against H/R-induced organoid injury in the co-culture systems were abolished by anti-IL-13.

### Interleukin-13 Promotes ISC Self-Renewal by Activating the Wnt or Notch Signaling Pathway During Intestinal I/R Injury

**Figure 7:**
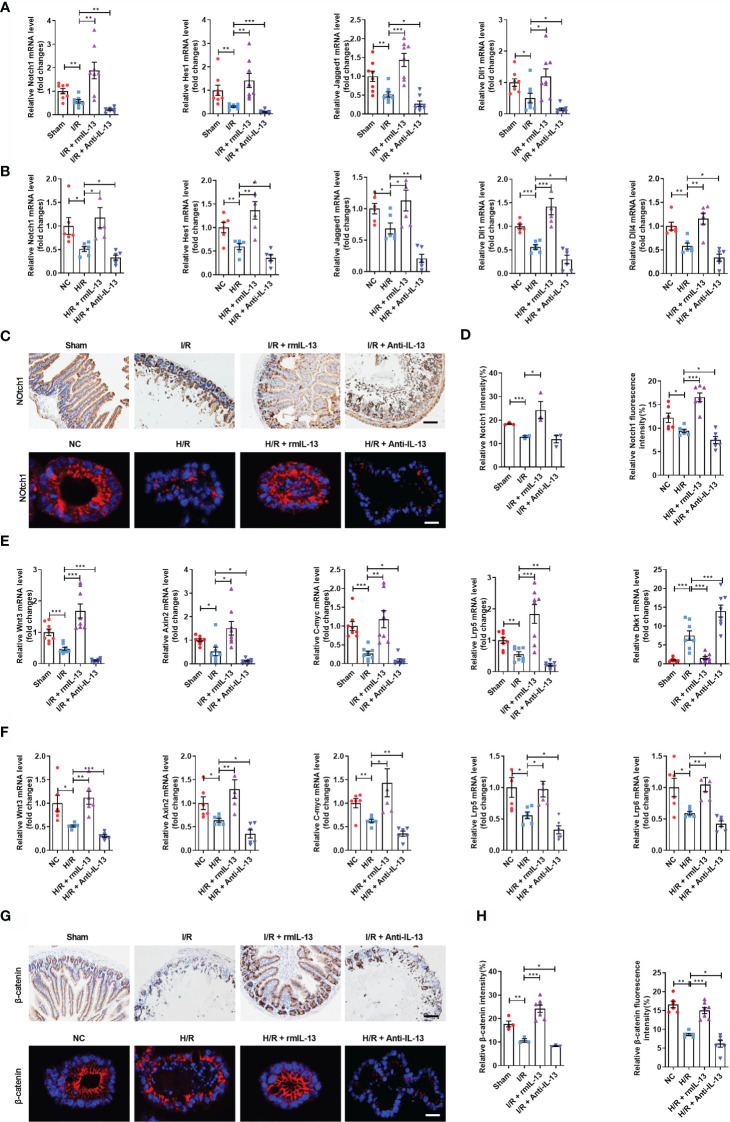
IL-13 promoted the self-renewal of ISCs by activating the Wnt or Notch signaling pathway during intestinal I/R injury *in vivo* and *in vitro*. **(A)** The mRNA levels of *Notch1*, *Hes1*, *Jagged1* and *Dll1* in the ileum were measured by quantitative PCR (n = 8). **(B)** The mRNA levels of *Notch1*, *Hes1*, *Dll1*, *Dll4* and *Jagged1* in the organoids were measured by quantitative PCR (n = 6). **(C, D)** Notch1 protein quantification analysis in the ileum (n = 8, scale bar is 100 μm) and organoids (n = 6, scale bar is 20 μm). **(E)** The mRNA levels of *Wnt3*, *Axin2*, *C-myc*, *Lrp5* and *Dkk1* in the ileum were measured by quantitative PCR (n = 8). **(F)** The mRNA levels of *Wnt3*, *Axin2*, *C-myc*, *Lrp5* and *Lrp6* in the organoids were measured by quantitative PCR (n=6). **(G, H)** Notch1 protein quantification analysis in the ileum (n = 8, scale bar is 100 μm) and organoids (n = 8, scale bar is 20 μm). The results are expressed as the mean ± SEM **(A, B, D, E, F, H)**. *p < 0.05, **p < 0.01, ***p < 0.001 by one-way ANOVA (Tukey’s test). PA, pravastatin; I/R, ischemia/reperfusion.

The Wnt/β-catenin and Notch signaling pathways are critical for ISC maintenance and self-renewal. We investigated the mechanism through which IL-13 promoted ISC self-renewal using rmIL-13 or anti-IL-13 antibodies. The mRNA expression encoding the Notch ligands *Jagged1*, *Dll1*, and *Hes1*, as well as the mRNA and protein levels of the Notch receptor *Notch1*, was lower in the I/R group than in the sham group (**Figures 7A, C, D**). The mRNA expression of the Notch ligands *Jagged1*, *Dll1*, *Dll4*, and *Hes1* and the mRNA and protein levels of the receptor *Notch1* in the H/R group were lower than those in the NC group (**Figures 7B–D**). These effects were enhanced by an anti-IL-13 antibody and reversed by rmIL-13. The mRNA expression of *Wnt3* (**Figure 7E**), Wnt receptor *Lrp5* (**Figure 7E**), Wnt target genes *C-myc* and *Axin2* (**Figure 7E**) and the protein expression of β-catenin were lower in the I/R group (**Figures 7G, H**), whereas levels of the Wnt antagonist *Dkk1* (**Figure 7E**) were higher than those in the sham group. The mRNA expression of *Wnt3* (**Figure 7F**), Wnt receptors *Lrp5* and *Lrp6* (**Figure 7F**), and the Wnt target genes *C-myc* and *Axin2* (**Figure 7F**) and the protein expression of β-catenin (**Figures 7G, H**) were lower in the H/R group than in the NC group. These changes were strengthened by the anti-IL-13 antibody and reversed by rmIL-13.

## Discussion

In the present study, we demonstrated for the first time that PA, identified as a metabolite of intestinal flora, attenuates intestinal I/R injury and improves the survival of animals, accompanied by promoting the self-renewal and proliferation of ISCs. Further, we showed that the protective effects of PA against intestinal I/R injury depends on the activation of ILC2s, mediated by IL-33/ST2 signaling. Interestingly, the current findings also indicated that the protective effects of PA or IL-33 against intestinal I/R injury depend on IL-13 released by activated ILC2s. Moreover, we found that IL-13 promoted ISC self-renewal to maintain the barrier homeostasis during intestinal I/R by activating Notch1 and Wnt signaling. Clinical evidence showed that the levels of PA in preoperative stool of patients undergoing CPB are negatively correlated with the parameters of postoperative intestinal injury, further suggesting that PA can reduce intestinal I/R injury.

Another study by our group confirmed that intestinal I/R leads to significant intestinal flora disorders and changes in metabolites (including capsiate and PA). The gut microbiota metabolite capsiate enhances Gpx4 expression and inhibits ferroptosis by activating TRPV1 in intestinal I/R injury. Based on two independent studies using the same research methods, PA and capsiate were both confirmed to be metabolites of intestinal flora, significantly reduce intestinal and organoid H/R injury, and be related to the level of postoperative intestinal I/R injury in clinical patients. The difference is that capsiate directly reduces intestinal I/R injury, but the PA-mediated reduction in intestinal I/R injury requires the interaction among intestinal epithelial cells, intestinal flora/metabolites, and immune cells. Moreover, as different drugs reduce intestinal I/R injury, their protective mechanisms are also completely different.

Some statins have been reported to reduce organ I/R injury. Simvastatin reduces subsequent intestinal tissue damage by suppressing oxidative stress, inflammatory damage, and apoptosis (37, 38). Atorvastatin has tissue-specific protective activity against intestinal I/R-induced injury (39). However, the role of PA in intestinal I/R injury remained obscure. Here, we confirmed that PA reduced intestinal I/R injury, maintained the intestinal mucosal homeostasis, and promoted ISC self-renewal. We also showed that the protective effects of PA against intestinal I/R injury depend on ILC2 activation *via* IL-33/ST2 signaling. Furthermore, data from patients showed that the levels of PA in preoperative feces of patients undergoing CPB were negatively correlated with the degree of postoperative intestinal I/R injury. Taken together, PA might be effective for treating intestinal I/R injury.

Interleukin-33 plays an important role in tissue repair and is mainly secreted by non-hematopoietic fibroblasts and epithelial and endothelial cells (12). Fluvastatin, simvastatin, atorvastatin, and lovastatin, but not PA, increase *IL-33* mRNA and intracellular IL-33 protein levels in both human adult cardiac myocytes and fibroblasts (13). In the current study, we showed that PA promoted the release of IL-33 by intestinal epithelial cells. Interleukin-33 might have therapeutic effects on ischemic stroke by promoting macrophage M2 polarization and cytokine production (40). The IL-33-ILC2 axis has a major protective role against renal I/R injury and thus could be a potential therapeutic strategy (29). However, others have also showed a harmful effect of IL-33 by promoting organ I/R injury. Promoting *IL-33* transcription in endothelial cells might contribute to I/R-induced renal injury and fibrosis (41). Interleukin-33 promotes kidney I/R injury *via* iNKT cell recruitment and cytokine production, thus resulting in neutrophil infiltration and activation at injury sites (42). Interleukin-33 promotes the formation of neutrophil extracellular traps, thus exacerbating liver I/R injury (43). The various roles of IL-33 in organ I/R injury might be due to differences in established models that use different organs or because IL-33 acts on different cells, such as ILC2 or neutrophils, to release different cytokines. However, the role of IL-33 in intestinal I/R injury has not been elucidated. ILC2s are innate producers of type 2 cytokines and key regulators of intestinal homeostasis. Depleting ILC2s, but not Tregs, substantially abolishes the protective effect of IL-33 on renal I/R injury (29). Meanwhile, the adoptive transfer of ILC2s reduces kidney I/R injury in mice (36, 44). However, the absence of ILC2s does not change the severity of kidney I/R damage (45). The role of ILC2s in intestinal I/R remained unknown. Here, we showed that the protective effects of PA against intestinal I/R injury depend on IL-33/ST2 signaling to activate ILC2s. Thus, we revealed the key role of IL-33/ST2 signaling and ILC2s in intestinal I/R injury. However, we also found that the absence of ILC2s does not change the severity of intestinal I/R injury. The reason for this phenomenon might be due to redundancy and compensation by other immune cells, such as Treg, AAM, and TH2 cells, as well as the decrease in the abundance of ILC2s during intestinal I/R. Since intestinal I/R is a model of severe acute injury, we measured mouse tissue 2 h after reperfusion. At this time, we found that the endogenous IL-33 level in the intestinal tissue was significantly reduced. Consistent with our results, Maroua Ferhat et al. showed a significant decrease in IL-33 1 h after kidney I/R compared to that in sham-group kidneys (42). Nozomu Sakai et al. found that IL-33 protein expression was decreased at 1 h after liver reperfusion, increased within 4 h after reperfusion, and remained elevated for up to 24 h (46). However, some studies have found that the release of endogenous IL-33 is increased in the early stage of reperfusion. The expression of IL-33 in oligodendrocytes and astrocytes increases rapidly after 60 min of transient middle cerebral artery occlusion (47). Furthermore, acute myocardial infarction leads to a significant increase in the release of IL-33 (48). Therefore, the difference in the level of endogenous IL-33 is not only related to the I/R injury of different organs and tissues but also the time taken for detection after reperfusion.

Interleukin-13 is a multifunctional cytokine secreted by ILC2s (19). It protects against intestinal warm I/R injury and plays a critical role in the regulation of Stat6 and Toll-like receptor-4 signaling (49). Consistent with these findings, we also confirmed that IL-13 reduced intestinal I/R injury. Furthermore, we revealed that PA increased the number of ILC2s and the proportion of IL−13^+^ ILC2s through the IL-33/ST2 axis, emphasizing the important role of IL-13 released by ILC2s during intestinal I/R injury. Interleukin-13, produced by ILC2s, promotes ISC self-renewal *via* the circular RNA circPan3 (19). However, the effect of IL-13 on ISCs and its underlying mechanism during intestinal I/R remained unclear. Here, we showed that IL-13 promotes ISC self-renewal by activating the Wnt/β-catenin and Notch pathways during intestinal I/R injury.

There are some limitations in this study. Although we demonstrated that PA is a metabolite of the gut microbiota, which can be produced by specific strains, how the PA-producing flora change during intestinal I/R and whether specific strains reduce intestinal I/R injury need further investigation. Although statins have been proven to promote the expression of IL-33 through epigenetic changes and other means (13), this research did not demonstrate how PA regulates IL33 release and whether PA directly regulates IL-33 during intestinal I/R injury. In addition, helminths, allergens, and certain protists induce type 2 immune responses (14). IL-33-mediated ILC2 expansion promotes the rapid activation of mast cells and the intestinal excretion of parasites (50). Products secreted by the mouse parasite *Heligmosomoides polygyrus* suppress type 2 (allergic) immune responses by interfering with the IL-33 pathway (51). PA changed the levels of IL-33, ILC2, and IL-13, but in this study, we did not consider the effect of PA on parasites. In addition, we referred to many references for the dosage of the anti-IL-33 and anti-ST2 neutralizing antibodies to ensure the neutralization efficiency while avoiding the toxic effect as much as possible. However, this research did not directly assess the neutralizing efficiency and toxicity of anti-IL 33 and anti-ST2 *in vivo*.

The present study demonstrated the protective effects of the intestinal flora metabolite PA on intestinal I/R injury, showed that PA promotes IL-13 release from ILC2s through IL-33/ST2 signaling, and confirmed the mechanism through which IL-13 promotes the self-renewal of ISCs. This study sheds light on a novel mechanism of intestinal I/R injury and provides a new therapeutic strategy for preventing intestinal I/R injury in a clinical setting.

## Data Availability Statement

The raw data supporting the conclusions of this article will be made available by the authors, without undue reservation.

## Ethics Statement

The studies involving human participants were reviewed and approved by Ethical Committee of Nanfang hospital, Southern Medical University. The patients/participants provided their written informed consent to participate in this study. The animal study was reviewed and approved by Committee of the Nanfang Hospital of Southern Medical University.

## Author Contributions

FD and K-XL conceived and designed the project. J-JH, B-CZ, and CL performed all clinal experiments. FD, J-JH, and XY performed all animal experiments and analyzed all animal data. Q-SS, Z-BL, Z-LC, YL and Z-ZY performed histology, PCR, intestinal permeability and ELISA experiments. W-FL and CL collected clinical samples and analyzed data. FD, J-JH, and K-XL wrote the paper with the assistance of the other authors. All authors contributed to the article and approved the submitted version.

## Funding

This work was supported by grants from National Natural Science Foundation, Beijing, China (81671955 to K-XL, 82902010 to J-JH), Key Program of National Natural Science Foundation, Beijing, China (81730058 to K-XL).

## Conflict of Interest

The authors declare that the research was conducted in the absence of any commercial or financial relationships that could be construed as a potential conflict of interest.

## Publisher’s Note

All claims expressed in this article are solely those of the authors and do not necessarily represent those of their affiliated organizations, or those of the publisher, the editors and the reviewers. Any product that may be evaluated in this article, or claim that may be made by its manufacturer, is not guaranteed or endorsed by the publisher.
