# Supplementary material for: Gut Microbial Metabolite Pravastatin Attenuates Intestinal Ischemia/Reperfusion Injury Through Promoting IL-13 Release From Type II Innate Lymphoid Cells via IL−33/ST2 Signaling
Source: Front Immunol. 2021 Sep 28;12:704836. doi: 10.3389/fimmu.2021.704836 (PMC8505964; doi:10.3389/fimmu.2021.704836)
Supplement: Supplementary file 1 [file DataSheet_1.pdf]

**Supplemental**

**Supplemental Table 1**

**Supplementary figure legends**

**Supplementary Table 1 Primers sequence of RT-PCR.**

| Gene | Forward primer (5'–3') | Reverse primer (5'–3') |
|---|---|---|
| 18S | CGATCCGAGGGCCTCACTA | AGTCCCTGCCCTTTGTACACA |
| ZO-1 | AGAGACAAGATGTCCGCCAG | TGCAATTCCAAATCCAAACC |
| Occludin | CATTTATGATGAACAGCCCC | GGACTGTCAACTCTTTCCGC |
| Ki67 | ATCATTGACCGCTCCTTTAGGT | GCTCGCCTTGATGGTTCCT |
| Lgr5 | CCTACTCGAAGACTTACCCAGT | GCATTGGGGTGAATGATAGCA |
| IL-33 | ATTTCCCCGGCAAAGTTCAG | AACGGAGTCTCATGCAGTAGA |
| ST2 | GAAGGCACACCGTAAGACTA | GACAAACCAACGATAGGAGG |
| IL-13 | TGAGCAACATCACACAAGACC | GGCCTTGCGGTTACAGAGG |
| Notch1 | GATGGCCTCAATGGGTACAAG | TCGTTGTTGTTGATGTCACAGT |
| Hes1 | TCAACACGACACCGGACAAAC | ATGCCGGGAGCTATCTTTCTT |
| Jagged1 | CCTCGGGTCAGTTTGAGCTG | CCTTGAGGCACACTTTGAAGTA |
| Dll1 | GCAGGACCTTCTTTCGCGTAT | AAGGGGAATCGGATGGGGTT |
| Dll4 | TTCCAGGCAACCTTCTCCGA | ACTGCCGCTATTCTTGTCCC |
| Wnt3 | TGGAACTGTACCACCATAGATGAC | ACACCAGCCGAGGCGATG |
| Axin2 | GGACTGGGGAGCCTAAAGGT | AAGGAGGGACTCCATCTACGC |
| C-myc | TTCATCTGCGATCCTGACGAC | CACTGAGGGGTCAATGCACTC |
| Lrp5 | AAGGGTGCTGTGTACTGGAC | AGAAGAGAACCTTACGGGACG |
| Lrp6 | TTGTTGCTTTATGCAAACAGACG | GTTCGTTTAATGGCTTCTTCGC |
| Dkk1 | CAGTGCCACCTTGAACTCAGT | CCGCCCTCATAGAGAACTCC |

**Supplementary figure legends**

**Supplementary figure S1 PA ameliorated mouse intestinal I/R injury and organoids H/R injury.** (A) Experimental design in vivo. The mice were randomly divided into the following groups. (1) Group sham operation group (sham group); (2) I/R group, in which SMA was occluded for 60 min followed by 2 h reperfusion; (3) I/R + PA group that was injected i.p. with 2 mg/kg PA 1 h before inducing intestinal I/R. (B-F) The relative mRNA levels of *IL-1β*, *IL-6*, *ZO-1*, *Occludin* and *Ki67* in the ileum were measured by quantitative PCR (n=8). (G) Experimental design in vitro. The WT monocultured organoids and the co-cultured organoid and ILC2 group were randomly assigned to a normal control (NC that was manipulated in the same manner as the H/R group but without undergoing H/R surgery); H/R group; H/R+PA group in which the organoids were incubated with 10 μmol/L PA 1 h before H/R. (H) The co-culture of small intestine organoids and ILC2 was observed under light microscope, scale bar is 50 μm, the black arrow indicates ILC2. (I-K) The relative mRNA levels of *ZO-1*, *Occludin* and *Ki67* in the organoids were measured by quantitative PCR (n=6). The results are expressed as the mean ± SEM (B-F, H-J). * $p < 0.05$, ** $p < 0.01$, *** $p < 0.001$ by one-way ANOVA (Tukey's test). PA, pravastatin; I/R, ischemia/reperfusion; SMA, superior mesenteric artery; WT, wild type; NC, normal control; H/R, hypoxia/reoxygenation; ILC2, type II innate lymphoid cells.

**Supplementary figure S2 PA protected against intestinal I/R injury via an IL-33/ST2 signal.** (A) Experimental design in vivo. The WT mice were randomly

divided into the following groups. (1) I/R group, in which SMA was occluded for 60 min followed by 2 h reperfusion; (2)I/R + PA group that was injected i.p. with 2 mg/kg PA 1 h before inducing intestinal I/R; (3)I/R + Anti-IL-33 group, in which WT mice were injected i.p. with 60 μg/kg Anti-IL-33 neutralizing antibody 2 h before establishing the intestinal I/R model; (4) I/R + PA + Anti-IL-33 group, in which WT mice were injected i.p. with 60 μg/kg Anti-IL-33 neutralizing antibody 2 h before intestinal I/R and 2 mg/kg PA 1 h before intestinal I/R in mice; (5) I/R + PA + Anti-ST2 group, in which WT mice were injected i.p. with 2 mg/kg PA and 1.5 mg/kg Anti-ST2 neutralizing antibody 1 and 2 h, respectively, before establishing the intestinal I/R model in mice. The IL-33$^{-/-}$ mice (IL-33$^{-/-}$) were randomly assigned to I/R and I/R + PA groups. (B-F) The relative mRNA levels of *ZO-1*, *Occludin*, *Ki67*, *IL-1β*, and *IL-6* in the ileum were measured by quantitative PCR (n=8). The results are expressed as the mean ± SEM (B-F). * $p < 0.05$, ** $p < 0.01$, *** $p < 0.001$ by one-way ANOVA (Tukey's test). PA, pravastatin; I/R, ischemia/reperfusion; SMA, superior mesenteric artery; WT, wild type.

**Supplementary figure S3 PA protected organoids H/R injury via an IL-33/ST2 signal.** (A) Experimental design in vitro. The cocultured WT organoid and ILC2 group were randomly assigned to (1) H/R group; (2) H/R + PA group; (3) H/R + Anti-IL-33 groups, in which co-culture was incubated with 1 ng/mL Anti-IL-33 neutralizing antibody 2 h before H/R; (4) H/R+PA+Anti-IL-33 group, in which co-culture was incubated with 1 ng/mL Anti-IL-33 neutralizing antibody 2 h before

intestinal I/R and 10 μmol/L PA 1 h before intestinal I/R in mice; (5) I/R+PA+Anti-ST2 group, in which co-culture was incubated with 10 μmol/L PA and 3 μg/mL Anti-ST2 neutralizing antibody 1 and 2 h before H/R, respectively. Meanwhile, co-cultured organoids from IL-$33^{-/-}$ mice and ILC2 from WT mice were randomly assigned to H/R and H/R + PA groups. (B-D) The relative mRNA levels of *ZO-1*, *Occludin* and *Ki67* in the organoids were measured by quantitative PCR (n=6). The results are expressed as the mean ± SEM (B-D). * $p < 0.05$, ** $p < 0.01$, *** $p < 0.001$ by one-way ANOVA (Tukey's test). PA, pravastatin; H/R, hypoxia/reoxygenation; WT, wild type; ILC2, type II innate lymphoid cells.

**Supplementary figure S4 Depletion of ILC2 abolished the protective effect of IL-33 on intestinal I/R injury and organoids H/R injury.** (A) Experimental design in vivo. The $Rag1^{-/-}$ mice were randomly divided into the following groups. (1) I/R group: $Rag1^{-/-}$ mice were injected i.p. with control rat IgG2b on days -5 and -2 before I/R); (2) I/R + rmIL-33 group: $Rag1^{-/-}$ mice were injected i.p. with control rat IgG2b on days -5 and -2 before I/R, and 50 μg/kg rmIL-33 2 h before intestinal I/R; (3) I/R + ILC2-/- group: $Rag1^{-/-}$ mice were injected i.p. with anti-CD90.2 antibody on days -5 and -2 before I/R surgery; (4) I/R + rmIL-33 + ILC2-/- group: $Rag1^{-/-}$ mice were injected i.p. with anti-CD90.2 antibody on days -5 and -2 before I/R surgery and 50 μg/kg rmIL- 33, 2 h before I/R. (B-F) The relative mRNA levels of *ZO-1*, *Occludin*, *Ki67*, *IL-1β* and *IL-6* in the ileum were measured by quantitative PCR (n=8). The results are expressed as the mean ± SEM (B-F). * $p < 0.05$, ** $p < 0.01$, *** $p < 0.001$

by one-way ANOVA (Tukey's test). PA, pravastatin; I/R, ischemia/reperfusion; WT, wild type; DTx, diphtheria toxin; WT, wild type; ILC2, type II innate lymphoid cells; rm, recombinant murine.

**Supplementary figure S5 Protection of organoids by IL-33 during H/R injury requires ILC2 participation.** (A) Experimental design in vitro. Organoids extracted from WT mice without ILC2 were randomly assigned to (1) H/R group; (2) H/R + rmIL-33 group. Meanwhile, co-culture system of organoids and ILC2 extracted from the WT mice were divided into 2 groups, H/R group and H/R + rmIL-33 group. (B-D) The relative mRNA levels of *ZO-1*, *Occludin* and *Ki67* in the organoids were measured by quantitative PCR (n=6). The results are expressed as the mean ± SEM (B-D). * $p < 0.05$, ** $p < 0.01$, *** $p < 0.001$ by one-way ANOVA (Tukey's test). PA, pravastatin; H/R, hypoxia/reoxygenation; WT, wild type; ILC2, type II innate lymphoid cells; rm, recombinant murine.

**Supplementary figure S6 PA/IL-33 protection against intestinal I/R injury was mediated by IL-13.** (A) Experimental design in vivo. The WT mice were randomly divided into the following groups. (1) I/R group; (2) I/R + PA group; (3) I/R + rmIL-33 group; (4) I/R + Anti-IL-13 group that was injected i.p. with 400 μg/kg Anti-IL-13 2 h before inducing intestinal I/R; (5) I/R + PA + Anti-IL-13 group; (6) I/R + rmIL-33 + Anti-IL-13 group. (B-F) The relative mRNA levels of *ZO-1*, *Occludin*, *Ki67*, *IL-1β* and *IL-6* in the ileum were measured by quantitative PCR (n=8). The

results are expressed as the mean ± SEM (B-F). * $p < 0.05$, ** $p < 0.01$, *** $p < 0.001$ by one-way ANOVA (Tukey's test). PA, pravastatin; I/R, ischemia/reperfusion; WT, wild type; rm, recombinant murine.

**Supplementary figure S7 Protection of organoids during H/R injury by PA/IL-33 was mediated by IL-13 released from ILC2.** (A) Experimental design in vitro. The co-cultured organoids and ILC2 extracted from the WT mice were randomly assigned to the following groups: H/R group, H/R + PA group, H/R + rmIL-33 group, H/R + Anti-IL-13 group, H/R + PA + Anti-IL-13 group and H/R + rmIL-33 + Anti-IL-13 group. (B-D) The relative mRNA levels of *ZO-1*, *Occludin* and *Ki67* in the organoids were measured by quantitative PCR (n=6). The results are expressed as the mean ± SEM (B-D). * $p < 0.05$, ** $p < 0.01$, *** $p < 0.001$ by one-way ANOVA (Tukey's test). PA, pravastatin; H/R, hypoxia/reoxygenation; WT, wild type; ILC2, type II innate lymphoid cells; rm, recombinant murine.
