# Supplementary figures and images for: Gut Microbial Metabolite Pravastatin Attenuates Intestinal Ischemia/Reperfusion Injury Through Promoting IL-13 Release From Type II Innate Lymphoid Cells via IL−33/ST2 Signaling
Source: Front Immunol. 2021 Sep 28;12:704836. doi: 10.3389/fimmu.2021.704836 (PMC8505964; doi:10.3389/fimmu.2021.704836)

Figure supplement 1

A

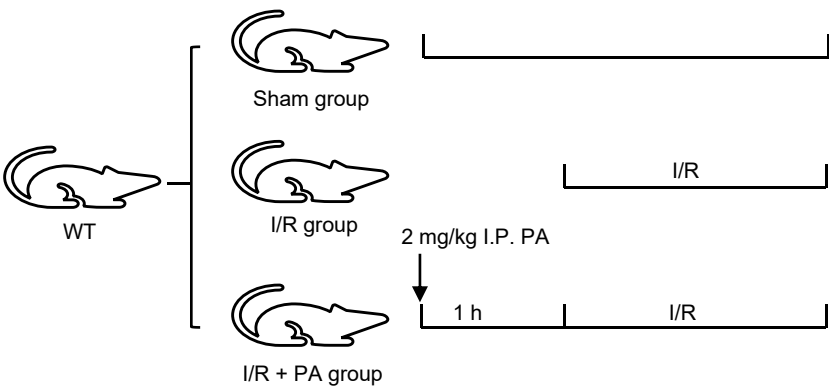

B

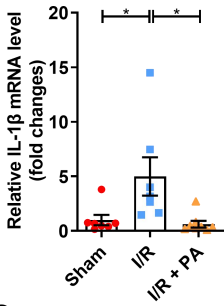

C

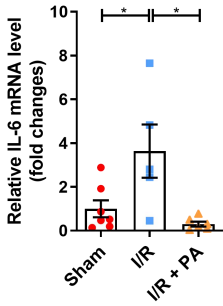

D

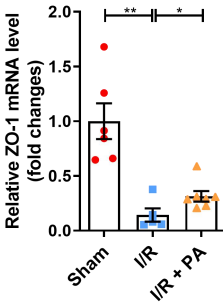

E

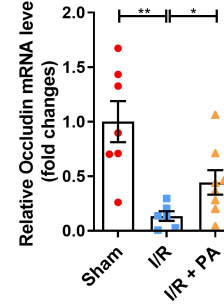

F

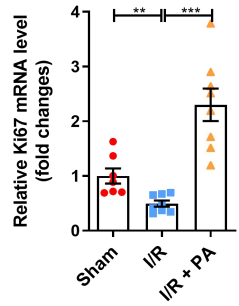

G

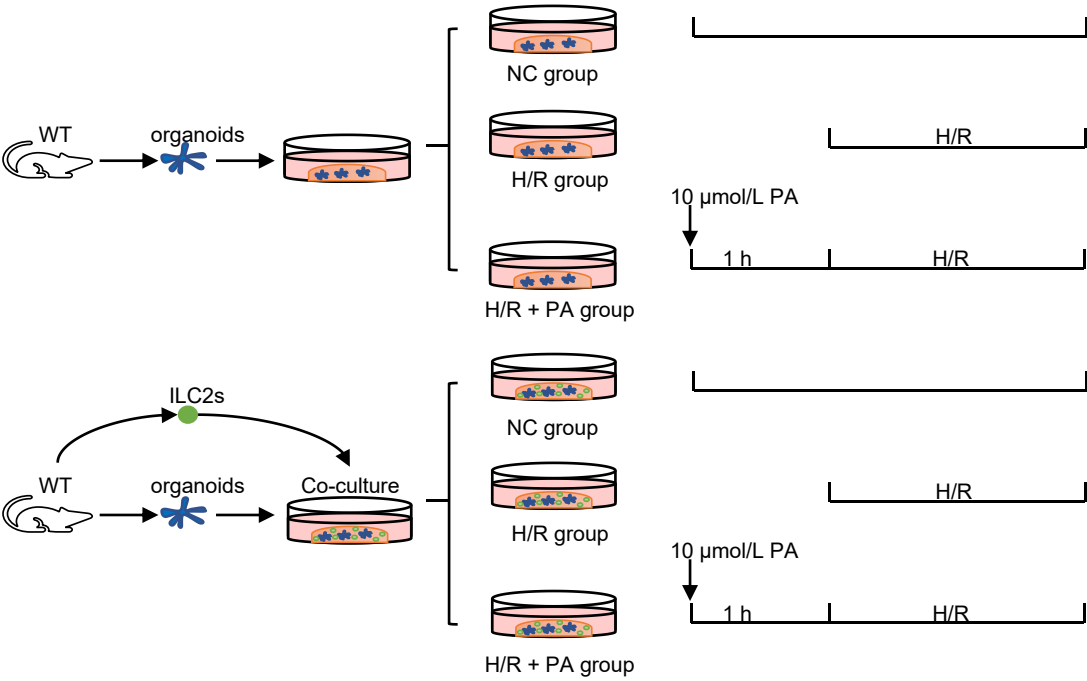

H

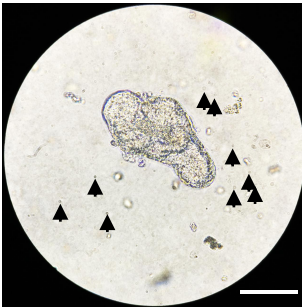

I

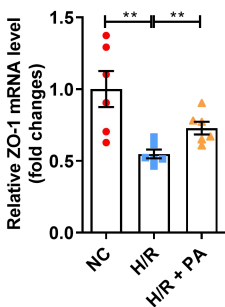

J

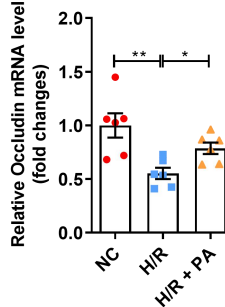

K

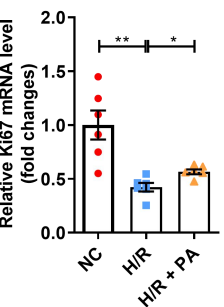

Supplement: Supplementary file 2 [file DataSheet_2.pdf]

Figure supplement 2

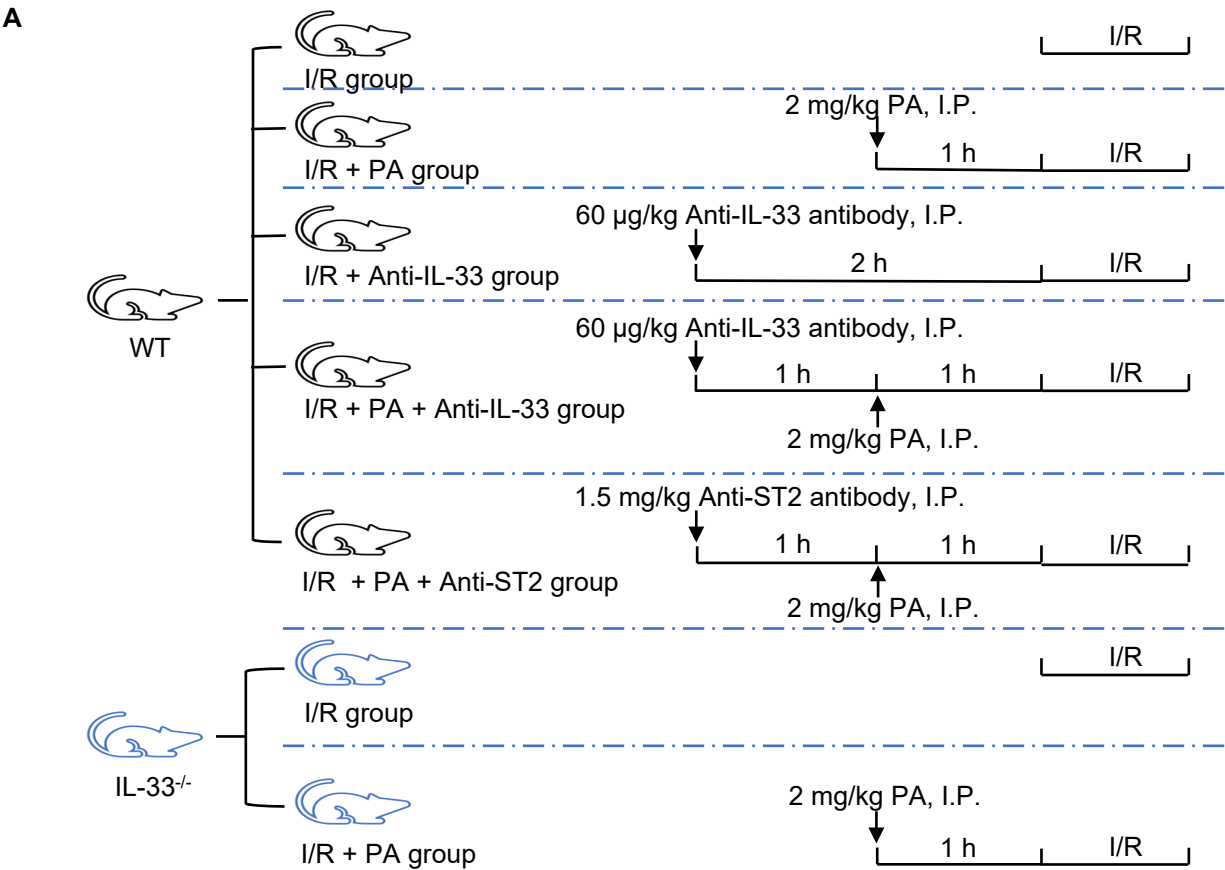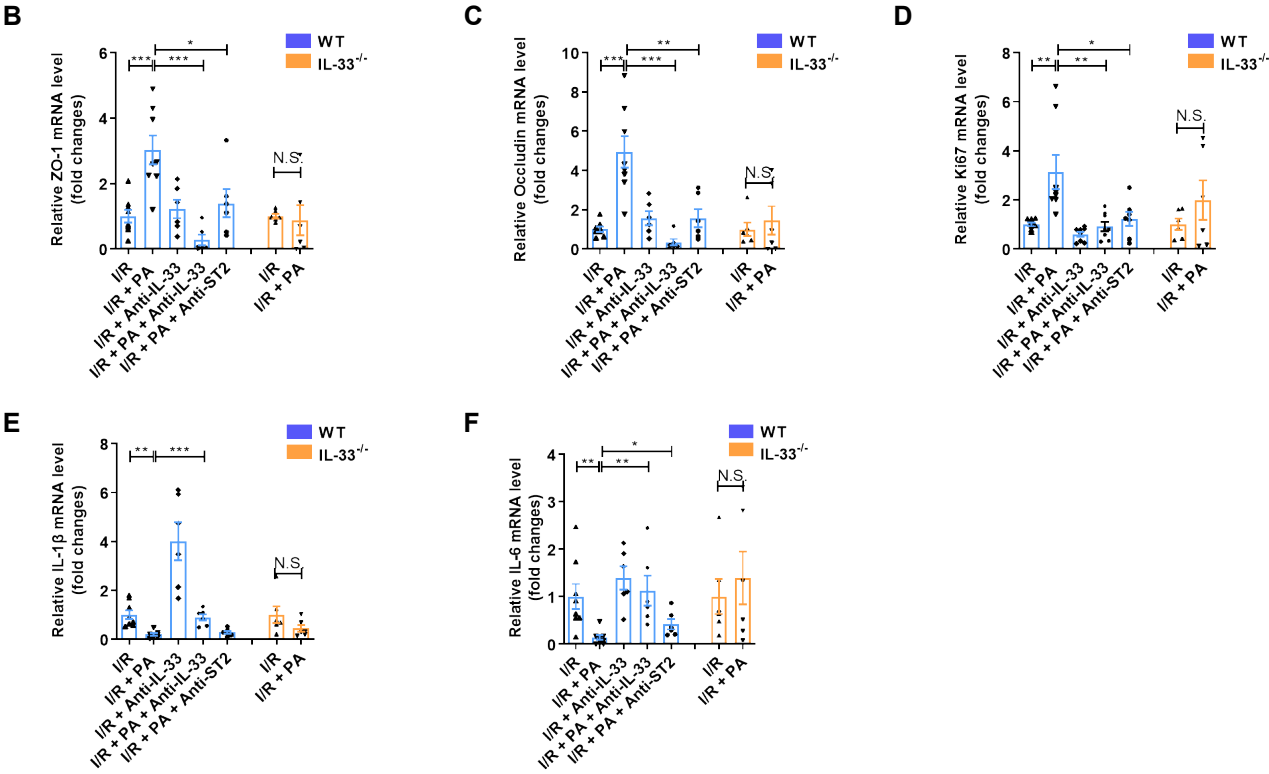

Supplement: Supplementary file 3 [file DataSheet_3.pdf]

Figure supplement 3

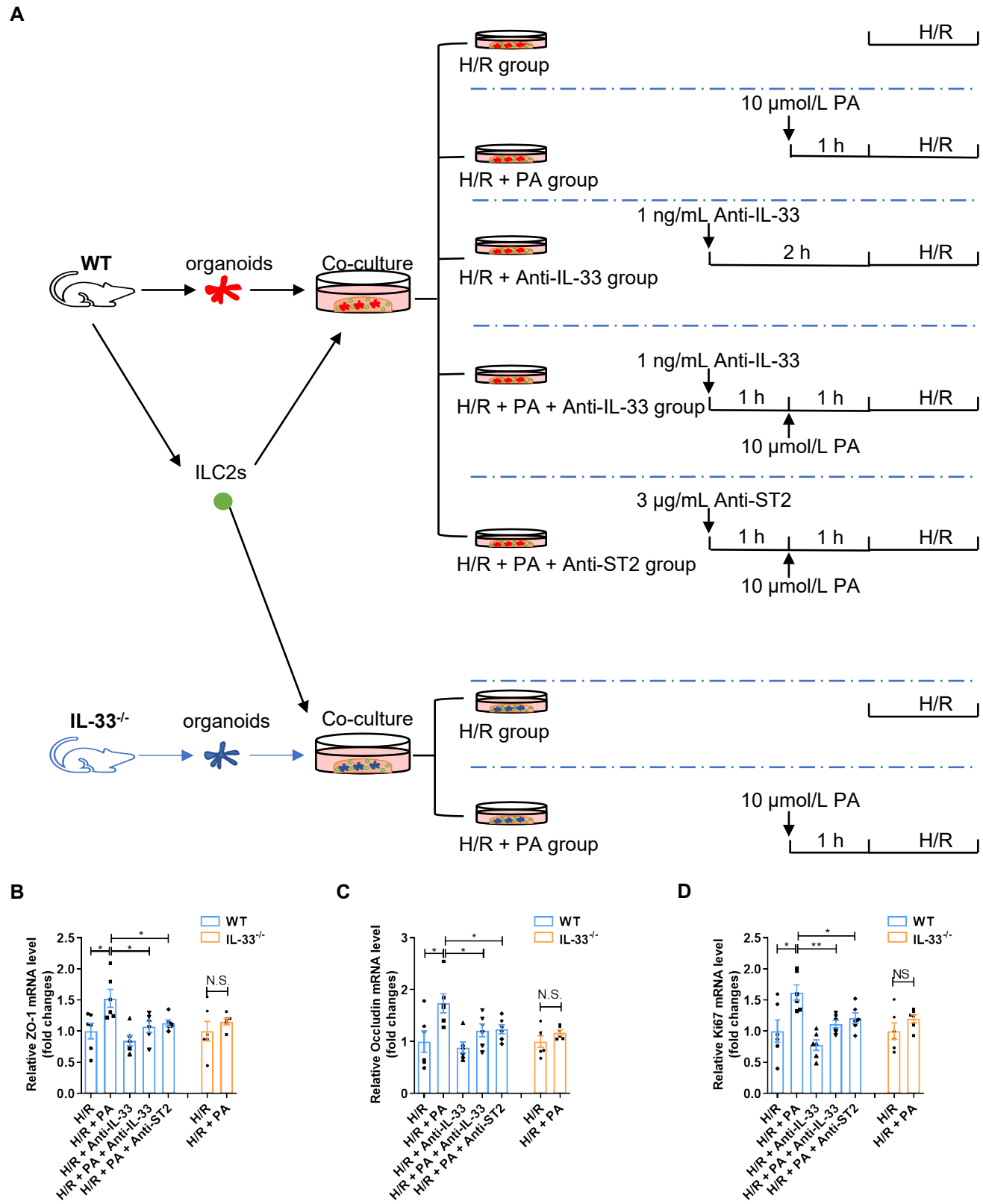

Supplement: Supplementary file 4 [file DataSheet_4.pdf]

Figure supplement 4

A

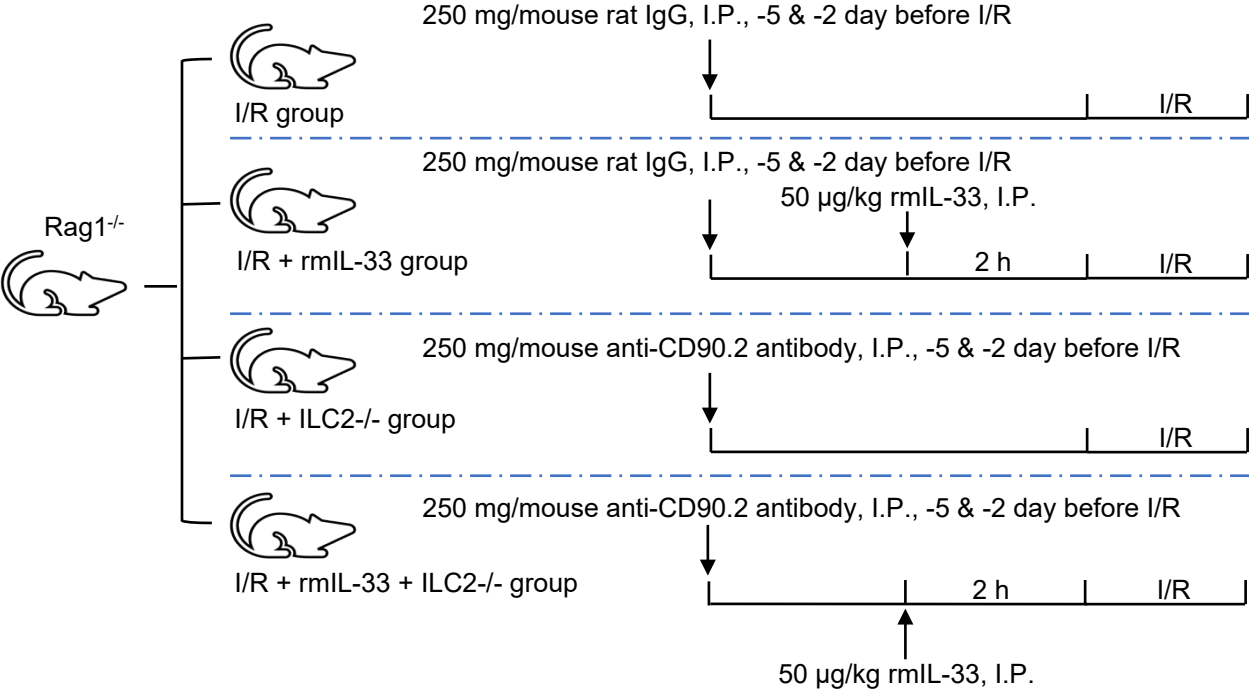

B

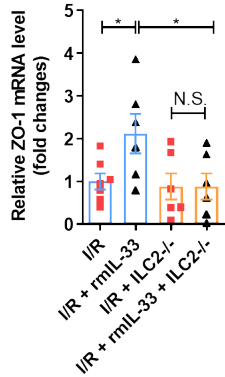

C

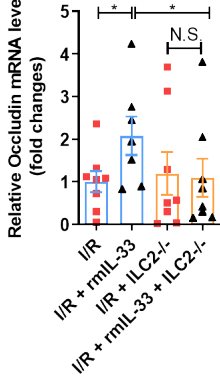

D

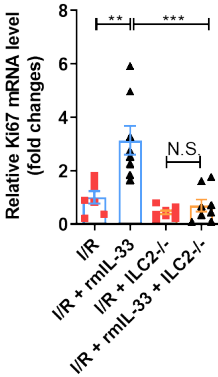

E

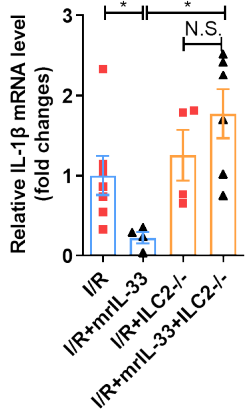

F

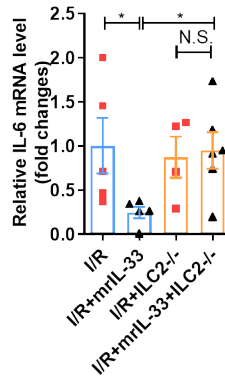

Supplement: Supplementary file 5 [file DataSheet_5.pdf]

Figure supplement 5

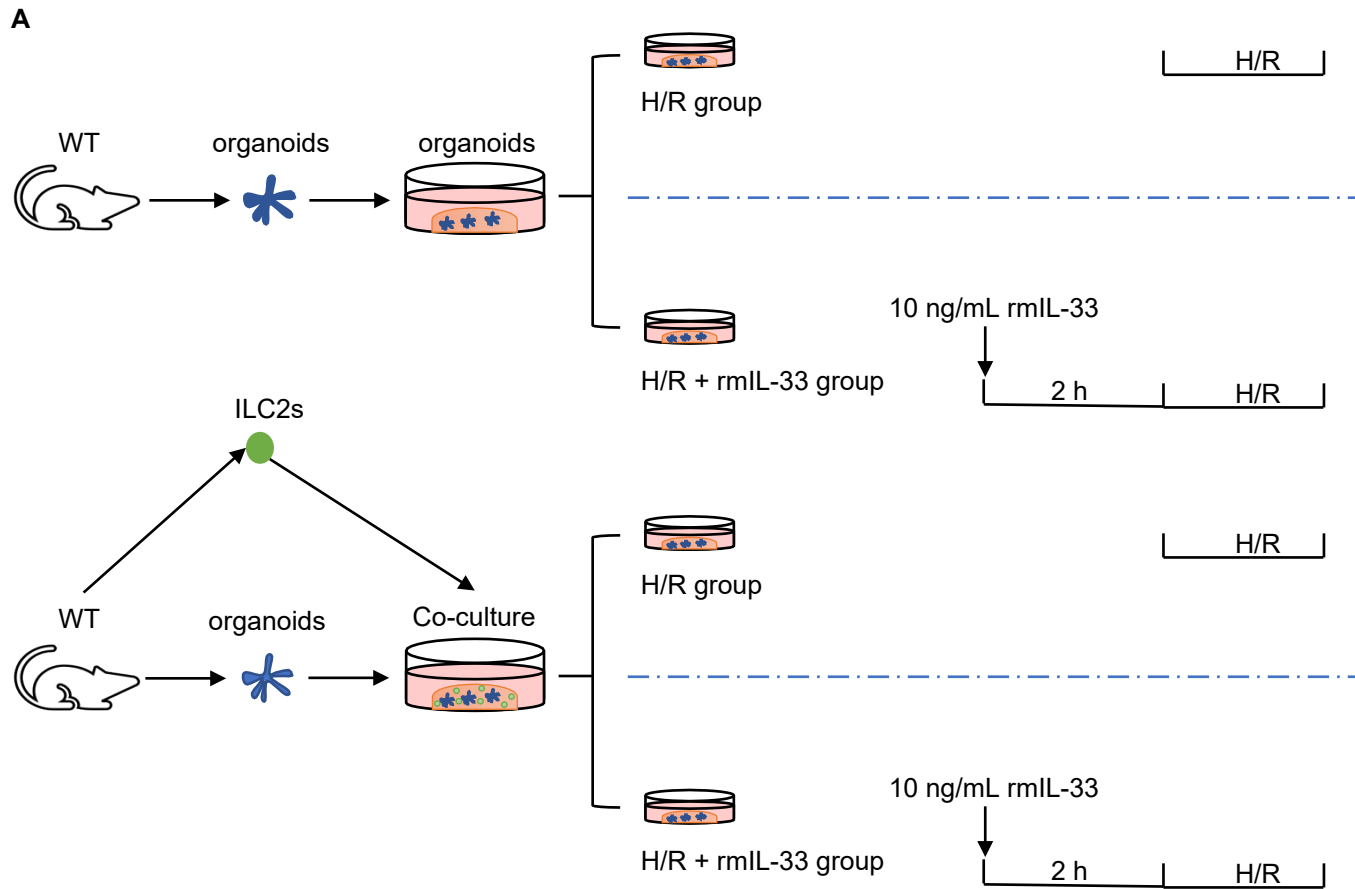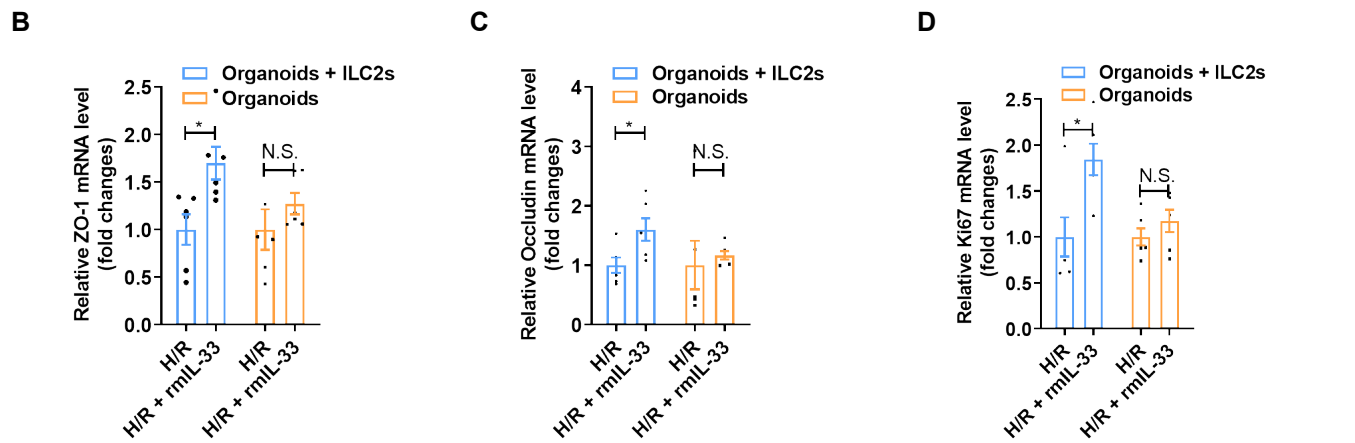

Supplement: Supplementary file 6 [file DataSheet_6.pdf]

Figure supplement 6

A

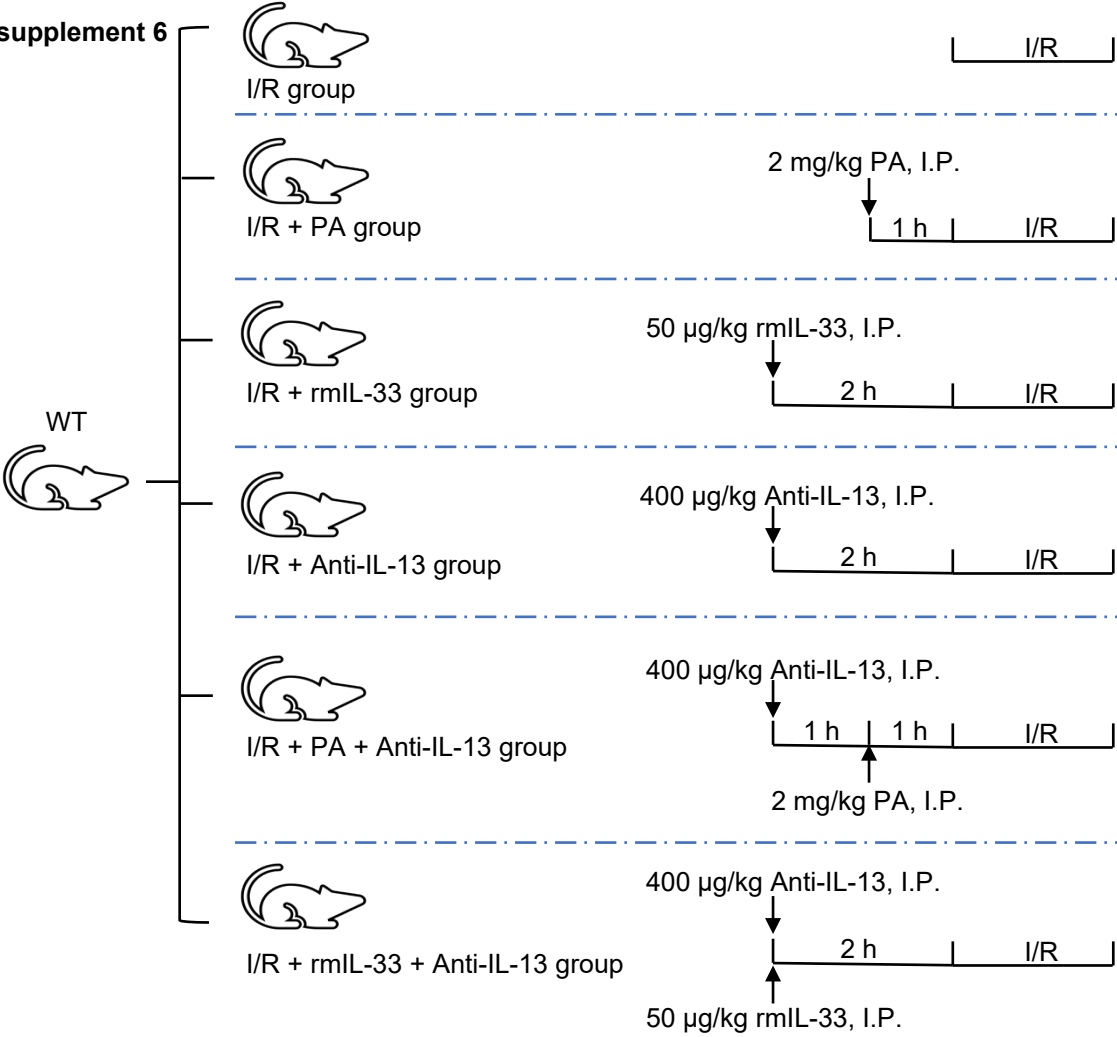

B

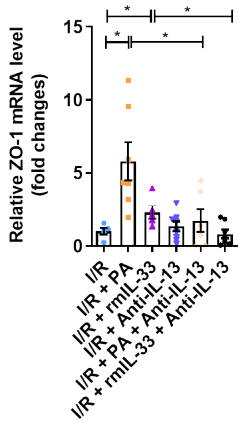

C

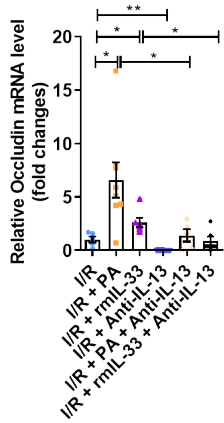

D

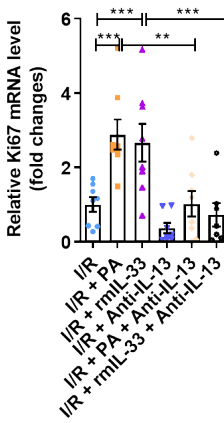

E

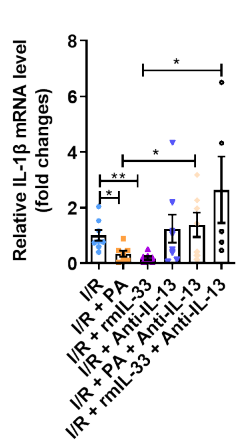

F

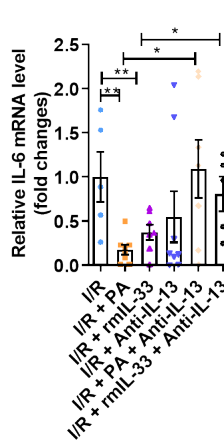

Supplement: Supplementary file 7 [file DataSheet_7.pdf]

Figure supplement 7

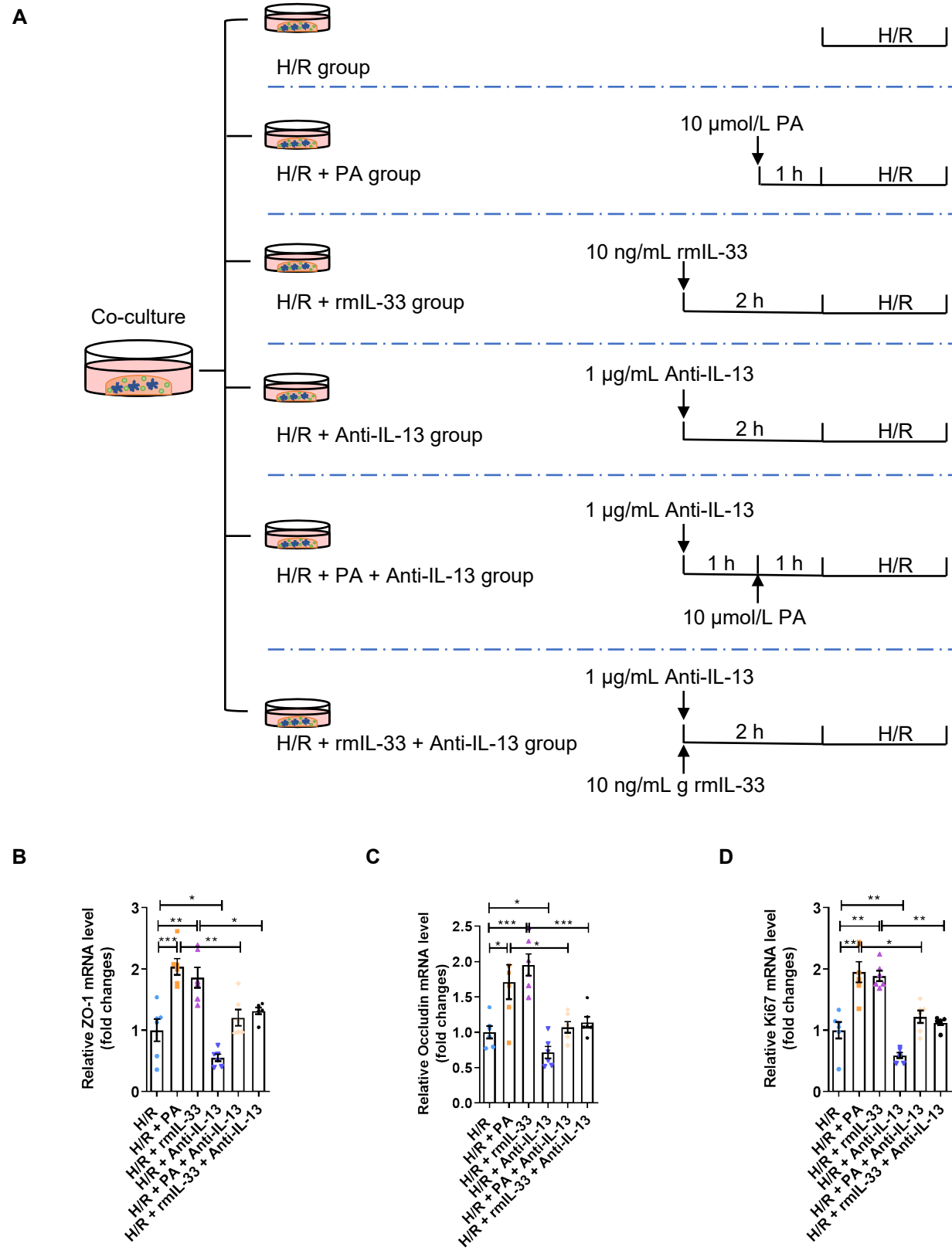

Supplement: Supplementary file 8 [file DataSheet_8.pdf]
